# Molecular-level enhanced clusterization-triggered emission of nonconventional luminophores in dilute aqueous solution

**DOI:** 10.1038/s41467-023-36115-w

**Published:** 2023-01-25

**Authors:** Qiuju Li, Xingyi Wang, Qisu Huang, Zhuo Li, Ben Zhong Tang, Shun Mao

**Affiliations:** 1 College of Environmental Science and Engineering, State Key Laboratory of Pollution Control and Resource Reuse, Tongji University, 1239 Siping Road, Shanghai, 200092 PR China; 2 School of Science and Engineering, Shenzhen Key Laboratory of Functional Aggregate Materials, The Chinese University of Hong Kong, Shenzhen City, Guangdong 518172 PR China

**Keywords:** Self-assembly, Organic molecules in materials science, Polymers

## Abstract

Nonconjugated and nonaromatic luminophores based on clustering-triggered emission derived from through-space conjugation have drawn emerging attention in recent years. The reported nonconventional luminophores are emissive in concentrated solution and/or in the solid state, but they tend to be nonluminescent in dilute solution, which greatly limits their sensing and imaging applications. Herein, we design unique clusteroluminogens through modification of cyclodextrin (CD) with amino acids to enable the intermolecular and intramolecular clusterization of chromophores in CD-based confined space. The resulted through-space interactions along with conformation rigidification originated from hydrogen bond interaction and complexation interaction generate blue to cyan fluorescence even in the dilute solution (0.035 wt.%, quantum yield of 40.70%). Moreover, the prepared histidine-modified CD (CDHis) is demonstrated for fluorescent detection of chlortetracycline with high sensitivity and selectivity. This work provides a new and universal strategy to synthesize nonconventional luminophores with bright fluorescence in dilute aqueous solution through molecular-level enhanced clusterization-triggered emission.

## Abstract (web summary)

Luminophores based on clustering-triggered emission have drawn emerging attention in recent years but they tend to be nonluminescent in dilute solution. Here, the authors design novel clusteroluminogens through modification of cyclodextrin (CD) with amino acids to enable clusterization of chromophores in CD-based confined space and realize blue to cyan fluorescence even in the dilute solution.

## Introduction

Organic fluorescent materials (OFMs), including small molecules and polymers, have been widely used in sensor, cell imaging, and display technology. Conventional OFMs built on through-bond conjugation (TBC) with large π-conjugated aromatic structures have tunable emission colors and high fluorescent efficiency^1–3^. However, these materials normally have poor water-solubility, high biotoxicity, high cost, and complex synthesis procedure, which greatly limit their practical applications^4^. In contrast, nonconventional luminophores are based on the clustering-triggered emission (CTE) effect and through-space conjugation (TSC) theory. Instead of aromatics, nonconventional luminophores possess nonconjugated structure based on saturated C–C, C–O, or C–N bonds and/or electron-rich heteroatoms, which have unique advantages of high biocompatibility, low toxicity, good processability, and facile preparation. Their unique luminescence behaviors and updated luminescence mechanism attract increasing attention in recent years^5–8^.

Nonconventional luminophores generally possess electron-rich heteroatoms, e.g., nitrogen (N), oxygen (O), sulfur (S), phosphorus (P), halogens (Cl, Br, and I), or subgroups containing C=O, C=C, and C≡N. They are emissive in concentrated solution and/or the solid state, but tend to be nonluminescent in dilute solution. This phenomenon is known as clusterization-triggered emission (CTE)^9–13^. The clusteroluminogens in aggregated state have been explored for encryption^14–17^ and bioimaging^18,19^. However, the concentration of clusteroluminogens used for cell imaging is 1000 times higher than traditional luminogens, which becomes a big obstacle for their practical applications^20^. Moreover, luminophores require effective interaction with analyte in sensing applications. The reported nonconventional luminophores are far from satisfactory as sensors due to the limited interaction between clusteroluminogens in solid state and analytes in aqueous solution^21,22^. Therefore, it is highly desirable to seek fresh strategy to promote the CTE effect for strong luminescence emission even in dilute solution.

The luminescence of mono-, di-, oligo-, and polysaccharides with abundant hydroxyl groups has been extensively studied as nonconventional luminophores^23–25^. They are weak emissive in dilute solutions, and bright emission is only observed from the concentrated solution (>8 wt.%) or crystalline state. In dilute solutions, linear polysaccharides chains, e.g., sodium alginate, adopt semi-rigid and show extended worm-like conformation, which results in non-luminescence due to the lack of sufficient electronic delocalization and active molecular motions^14^. Therefore, exploiting nonlinear molecules and enhancing molecular packing is crucial significance to obtain bright luminescence in dilute solution.

Cyclodextrin (CD) is comprised of D-glucopyranoside units linked together by α-1,4-glycosidic bonds, which has a well-formed shallow truncated cone^26–28^ The intrinsic cyclodextrin solution is nonluminescent due to the lack of clusterization of electron-rich moieties and consequent electron delocalization. However, the space-confined structure of CD molecule is a perfect platform to construct CTE in dilute solution. On the other hand, the luminescence of natural proteins was previously believed due to the aromatic amino acids (e.g., Trp, Tyr, and Phe). However, the intrinsic visible emission of nonaromatic amino acids^29^ and polypeptide^30^ were only observed in concentrated solution and solid phase, which was due to the CTE mechanism from nonconventional chromophores (i.e., amino, carbonyl, and hydroxyl) and subsequent electron cloud overlap with simultaneous conformation rigidification. However, neither of the inclusion complexes between CD and amino acid based on host-guest interaction^31–36^ nor amino acid-modified CD^37,38^ exhibits new fluorescence due to the inefficient CTE effect. The host-guest interactions between CD and aromatic amino acids including tryptophane, phenylalanine, and tyrosine may cause an increase in the fluorescence intensity, but cannot change the shape or position of the emission band^31–35^. For non-fluorescent amino acids such as l-arginine and l-lysine, no emerging fluorescent emission is observed^36^.

**Fig. 1:**
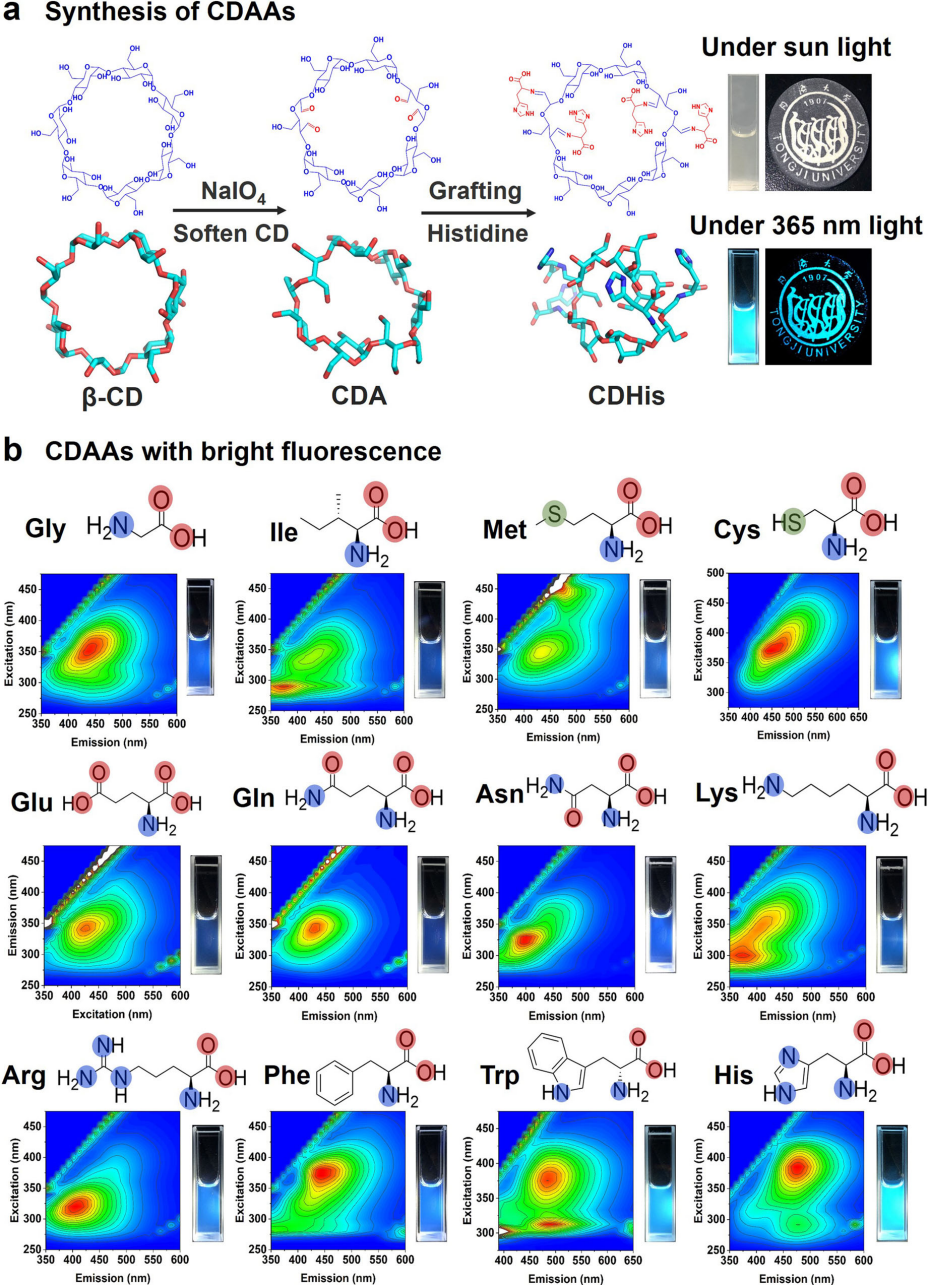
Synthesis of highly luminescent CDAAs. **a** Synthetic route of luminescent CDAAs (histidine as example) from the conjunction of β-CD and amino acid. Right: photos of CDHis in aqueous solution (1.0 mM in H_2_O) and powder phase under sunlight and 365 nm UV light, respectively. **b** The EEMs (excitation–emission matrix spectra) and fluorescent photos of CDAA solutions (1.0 mM in H_2_O) under 365 nm UV light.

Inspired by the unique structure of CD and CTE phenomenon in amino acids (AAs), we report a new strategy to enable the enhanced CTE of nonconventional chromophores in dilute solution. This is a pioneer report of nonconventional luminophores with strong luminescence in dilute solution through modification of cyclodextrin with amino acids. As shown in Fig. 1, partially-oxidized β-cyclodextrin aldehyde (CDA) with sodium periodate is firstly obtained, which exhibits high aqueous solubility and reactivity with functional amino groups. The oxidation of CD leads to the cleavage of C2–C3 bond and the collapse of CD skeleton, softening the initial rigid β-CD molecule. Then, 12 amino acids including nonaromatic amino acids and aromatic amino acids are grafted to the flexible CDA molecule. Taking histidine (His) as example, the amino acid-modified CD (CDAA) presents unique clusteroluminescence even in dilute solution (as low as 0.035 wt.%).

Different from the recrystallized amino acids with luminescence in aggregated states, the CDAAs endow the amino acids aggregating in the limited space from CD in sole molecular level in dilute solution. The strategy in this work is also different from the well-established luminescent host-guest complexes based on macrocycles such as CDs and curcubiturils. The host-guest interaction will cause the alteration of the excited state photophysics, such as the molecular rotor 9-(dicyano-vinyl)julolidine in CDs with different cavity-size^39^, and the change of the emission of fluorescent compounds when interacting with curcubiturils^40,41^. However, the supermolecular host-guest interactions are located in the cavity of macrocycles, causing limited intermolecular interaction of guest molecules^42^. Comparatively, in the current work, the nonconventional luminophores (amino acids) are chemically grafted to CD rather than forming an inclusion complex. This grafting further alters the structural rigidity of CD, which promotes the intermolecular interactions needed for CTE. The nonconventional fluorescence of CDAAs is attributed to enhanced intermolecular and intramolecular clusterization of chromophores including –COOH, –OH, and –NH in CD-based confined space and simultaneous conformation rigidification due to rich hydrogen bonds in CDHis. For other CDAAs obtained under alkaline condition, strong complexation interactions between the metal cation and carboxylic anions also increase its structural rigidity, favoring the fluorescence emission. The enhanced CTE effects based on conjunction of AAs moieties in CD skeleton in molecule designing enable the bright emission in dilute solution. With the unique nonconventional fluorescence in dilute solution, the application of this newly prepared CDAA as fluorescent probe for highly sensitive and selective detection of tetracycline antibiotic is also demonstrated.

This work provides an universal strategy to synthesize nonconventional luminophores based on amino acid-modified cyclodextrin, which exhibits bright fluorescence in dilute solution due to enhanced CTE mechanism. The reported simple and efficient strategy is believed to significantly promote the synthesis and broad applications of nonconventional luminophores in aqueous phase.

## Results

### Nonconventional luminescence of CDAAs

As shown in Fig. 1a, at first, β-cyclodextrin aldehyde (CDA) was obtained through the selectively oxidization of β-cyclodextrin with NaIO_4_, which led to the collapse of the cyclodextrin skeleton. According to the results of geometry optimization calculations, the CDA molecule exhibits shrunken cavity (Supplementary Fig. 1) and flexible cyclic skeleton. The FTIR and ^1^H NMR spectra of CDA are shown in Supplementary Figs. 2 and 3, which confirm the existence of aldehyde groups in CDA molecules. Secondly, common amino acids including nonaromatic amino acids and aromatic amino acids are grafted to CDA molecules through a Schiff-base reaction between the aldehyde groups in CDA and amino groups in histidine (His) (taking histidine as example). The characterization data including the ^13^C solid-state NMR spectra (Supplementary Figs. 4–6), the ^1^H NMR spectra (Supplementary Figs. 7 and 8), the FTIR spectra (Supplementary Fig. 9), and the N 1*s* and C 1*s* XPS spectra (Supplementary Figs. 10 and 11) confirm the successful conjunction of AAs and CDA through Schiff-base bonds. As shown in Supplementary Fig. 12, the lyophilized powder of CDAAs shows amorphous structure, which is different from the complexes formed by AA and CD based on host and guest interactions. Consequently, the His moieties are confined in the limited space in cyclodextrin, which leads to the through-space interactions and enhanced clusteroluminescence. The fluorescence images of CDHis in aqueous solution (1.0 mM) and in solid phase under 365 nm UV light were recorded, which showed bright fluorescence in dilute solution and highly luminous patterns in powder form (Fig. 1a).

The CDAA molecules, from conjunction of CD with nonaromatic amino acids (glycine, isoleucine, methione, cysteine, glutamic acid, glutamine, aspatagine, lysine, and arginine), aromatic amino acids (phenylalanine and tryptothan), and heterocyclic amino acid (histidine), have abundant electron-rich heteroatoms such as N, O, and even S. Consequently, the through-space n···n and through-space n···π interactions lead to the abnormal clusteroluminescence. The excitation–emission matrix spectra and fluorescent photos of CDAAs are shown in Fig. 1b. The CDAAs solutions exhibit bright blue to cyan fluorescence with emission light from 350 nm to 550 nm. The fluorescence photographs of CDAAs show bright fluorescence in dilute solution even under non-optimized excitation wavelength (365 nm), further confirming the high fluorescence properties of CDAAs. The results indicate the feasibility of the universal strategy to synthesize series of nonconventional CDAAs with bright fluorescence in dilute solution.

**Fig. 2:**
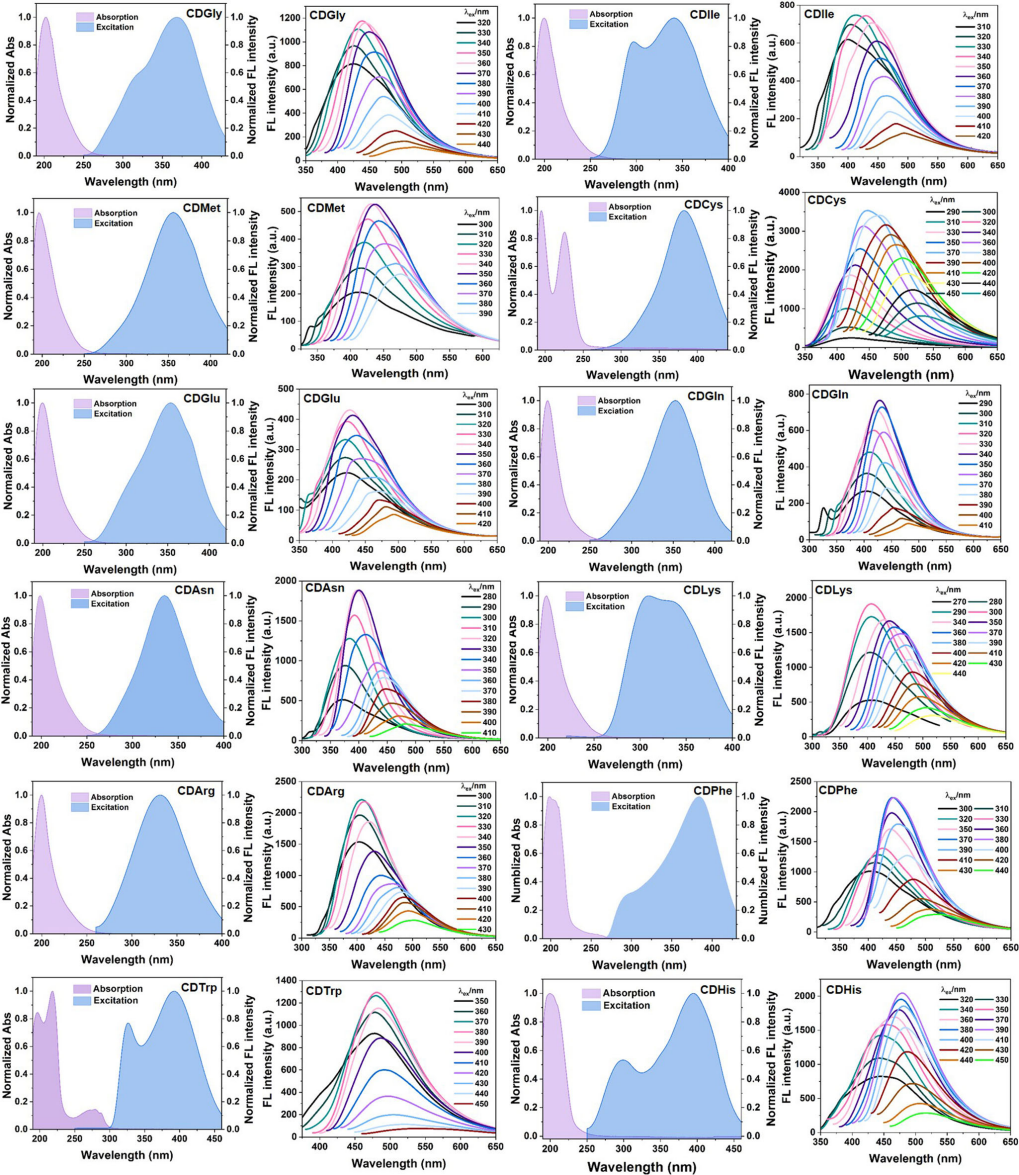
The unique fluorescence properties of CDAAs. Normalized UV–Vis absorption spectra, excitation spectra, and fluorescence spectra at different excitation wavelengths of CDAAs including CDGly, CDIle, CDMet, CDCys, CDGlu, CDGln, CDAsn, CDLys, CDArg, CDPhe, CDTrp, and CDHis. All the CDAA solutions were prepared in H_2_O with a concentration of 1.0 mM.

The absorption and excitation spectra of CDAAs in H_2_O were recorded to further study the fluorescence properties of CDAAs. As shown in the UV–Vis absorption spectra (Fig. 2), the maximum absorption peak locates at around 200 nm, which corresponds to the hydroxy units inside the CD. No typical absorption peaks for π-conjugated aromatic structures were observed, suggesting the nonconjugated nature that unlike traditional OFMs. Obviously, the absorption and excitation spectra show huge differences in the longer wavelength range, which is a general feature for clusteroluminescence^43^. The CDAAs could be excited by the light with a wavelength longer than 200 nm. Previous studies^44,45^ have proved that the longer excitation peak above 300 nm from the n–π* transition also exists in the absorption spectra, but the corresponding transition is forbidden. Therefore, this band is indetectable in the low-sensitivity absorption characterization, but is found in the hypersensitive excitation measurement.

The fluorescence spectra of each CDAA solution were recorded at different excitation wavelengths (λ_ex_) from 290 to 440 nm. The maximum emission peak (λ_em_) is red-shifted from 400 nm to 530 nm along with the increase of λ_ex_. The excitation-dependent fluorescence is also one of the normal features of nonconventional luminophores in concentrated solution or crystals state^9,25,46^. Such excitation-dependent emission is attributed to the diversified emission species with different effective through-space conjugations of AAs moieties grafted on the CD skeleton, which is essentially resulted from the heterogenous clustering of functional moieties including C=N, N–H, C=O, and OH groups. Therefore, it is also highly feasible to regulate the fluorescence by varying the excitation wavelength.

### Luminescent mechanism of CDAAs

**Fig. 3:**
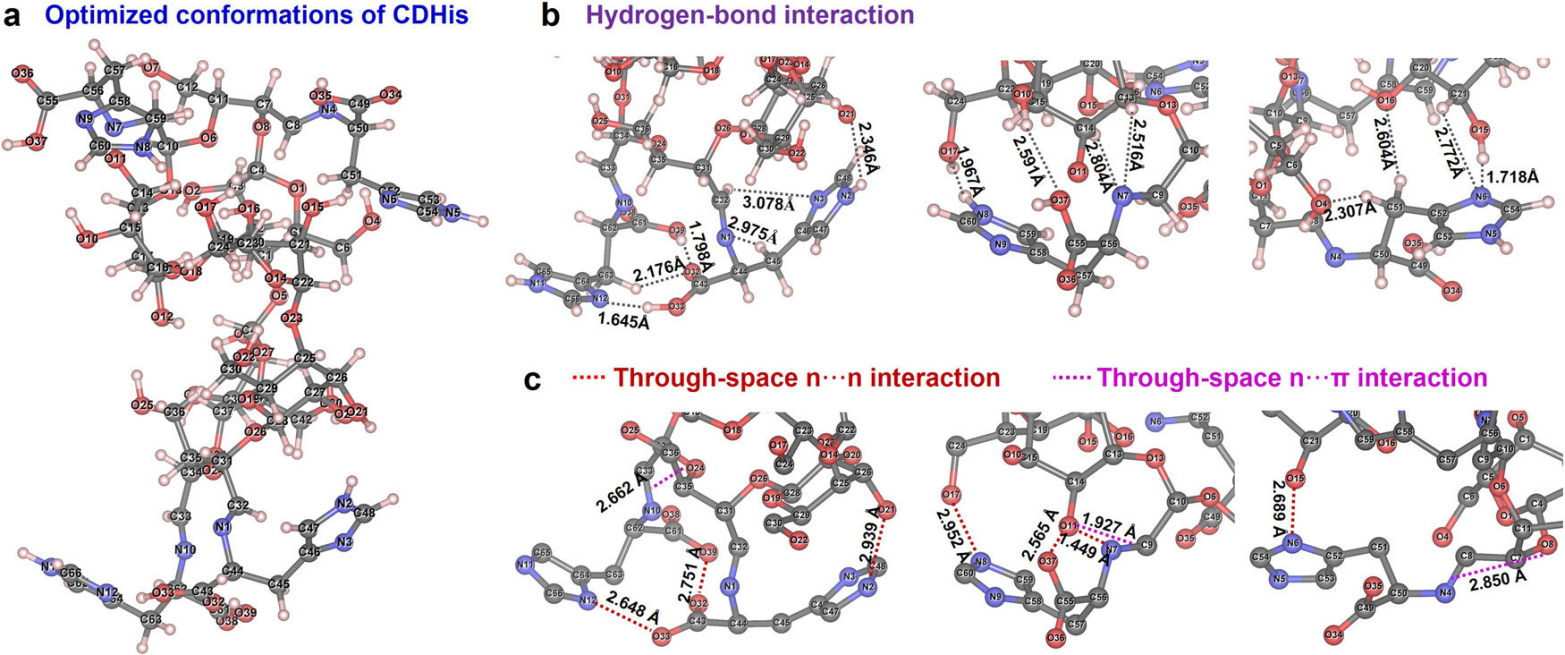
The intramolecular interactions in CDHis. **a** Optimized conformations of CDHis. **b** Hydrogen bonds interaction around His moieties. **c** Through-space n···n and n···π interactions between His moieties and cyclodextrin skeleton.

Taking CDHis with the brightest luminescence as example, the fluorescence intensity increased with the increasing amount of histidine (Supplementary Fig. 13), which indicates the contribution of His moieties on the emission intensity. The electrospray ionization mass spectrometry (ESI-MS) analysis (Supplementary Fig. 14) confirm the successful grafting of histidine onto CDA through the Schiff-base reaction with the stoichiometric ratio of 1:4 (CD:His). The high-resolution ESI mass spectra of the other CDAAs (Supplementary Fig. 15) and the fluorescence intensity variations with the increasing amount of AAs (Supplementary Fig. 16) together confirm the stoichiometric ratio (CD:AA) of 1:2 in CDTrp, 1:3 in CDAsn, CDCys, CDGln, and CDLys, and 1:4 in CDGlu, CDGly, CDIle, CDMet, CDPhe, and CDArg. Among the 12 kinds of CDAAs, CDHis with 12 N atoms and 39 O atoms exhibit more bright fluorescence probably due to higher electron density with rich electrons in adjacent iminazole groups. The quantum yield of CDHis powder is determined as 7.8%, which is a little higher than those of the common amino acid crystals^29^. Particularly, the CDHis in dilute solution exhibits high quantum yield of 40.70% at 200 μM (0.035 wt.%) and 34.22% at 3 mM (0.525 wt.%), which are much higher than those of the reported nonconventional luminophores including concentrated amino acid and alginate solutions (Supplementary Fig. 17)^14,29^. To further depict the through-space conjugation and clusterization of heteroatoms in CDHis, geometry optimizations of CDHis were performed using the semi-empirical xtb program. By applying the large models (>340 atoms), it is found that the soften skeleton of CDA molecule further transforms in CDHis structure, and the His moieties are restricted in the confined space (Fig. 3a). In addition to the abundant O–H⋯O and C–H⋯O hydrogen bonds in cyclodextrin skeleton, a large number of O–H⋯N, C–H⋯O, C–H⋯N, N–H⋯O and O–H⋯O hydrogen bond interactions exist between His moieties and cyclodextrin skeleton (Fig. 3b), which contribute to the conformation rigidification of CDHis and facilitate the intermolecular electronic communication^47^.

For the other soft amino acids including glycine, isoleucine, methionine, cysteine, glutamic acid, glutamine, asparagine and phenylalanine, fluorescent CDAAs could be obtained only in alkaline solution. This is possibly because strong complexation interaction between the metal cation and carboxylic anion in above amino acids will form a framework structure in CDAAs and increase its structural rigidity, resulting in the fluorescence emission^48^.

Through-space interaction has been identified as the behind mechanism of clusteroluminescence^43^. The O···O distance in cyclodextrin structure (Supplementary Fig. 18) lies in the range of 2.1–2.9 Å, which is shorter than the sum of the van der Waals radius of two oxygen atoms (3.04 Å)^49,50^. The through-space O···O interactions are linked in the CD-based space and form an n-electron band, favoring the extended electron delocalization. Moreover, the formation of Schiff-base linkage (C=N) between His moieties and cyclodextrin skeleton contributes more electron-rich nonconventional luminophores and simultaneously stiffens the molecular conformation through crosslinking and noncovalent intra/intermolecular interactions. Thus, this process enables effective clusterization and significantly boosts emission^9^. As shown in Fig. 3, the through-space n···n interaction (O···O or N···O) and n···π interaction ((n)O···C=N(π)) between His moieties and CD skeleton can be regarded as the emitting sources of nonconjugated clusteroluminogens, which are widely present in confined space of CD skeleton. On the hand, the C=N···O=C (π···π) interaction in His structure works as an effective through-space electronic communication channel, which generates optically excitable conjugates with rigidified conformation, thus offering bright visible emission upon UV excitation.

**Fig. 4:**
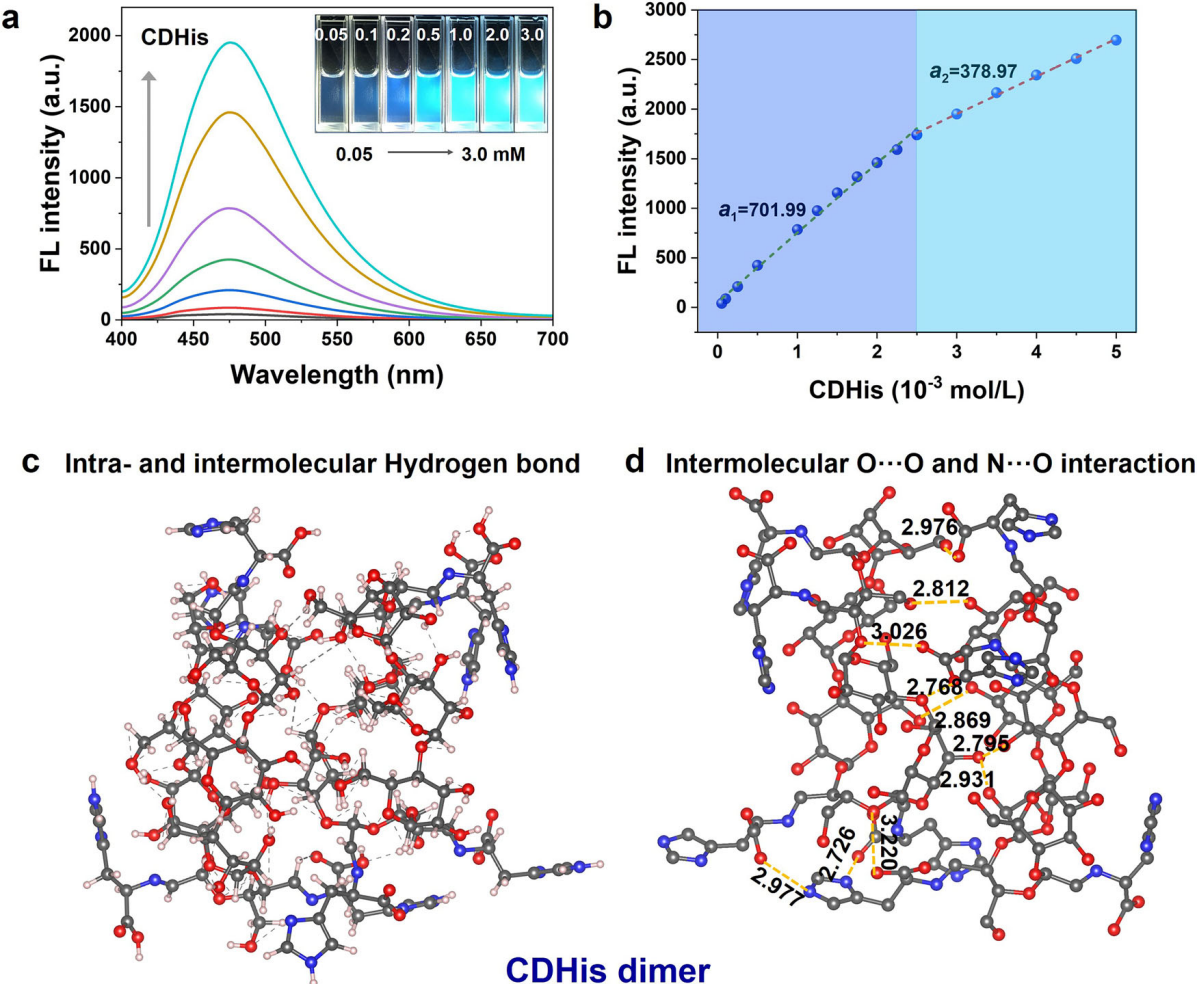
Fluorescence and intermolecular interactions of CDHis in dilute solution. **a** Concentration-dependent FL spectra of CDHis in aqueous solution (λ_ex_ = 390 nm). The inset shows the photographs of CDHis solutions under 365 nm UV light. **b** Plots of FL intensity vs. CDHis concentration in aqueous solution (λ_ex_ = 390 nm). **c** Intra- and intermolecular hydrogen binds in CDHis dimer. **d** Intermolecular O···O and N···O interactions in CDHis dimer.

To investigate the unconventional emission of CDHis in dilute aqueous solution, the concentration-dependent FL spectra of CDHis solution was taken (Fig. 4a). The inset of Fig. 4a shows the images of CDHis solutions with different concentrations. The FL intensity increases when the concentration of CDHis increases from 10^−5^ to 10^−3^ M, and the CDHis solution displays increasing bright luminescence, similar to the aggregation-induced emission (AIE) effect. It is worth noting that the bright emission could be observed from the diluted solutions with a concentration as low as 2 × 10^−4^ M or 0.035 wt.%, which is the unprecedented reported nonconventional chromophores with strong luminescence in dilute solution. In contrast, the solutions of CDA and His are not emissive under the same condition.

The FL intensity was plotted vs. the CDHis concentration. As shown in Fig. 4b, it is found that the fitting of FL intensity vs. CDHis concentration exhibits two different stages, where the *a*_*1*_ slope (701.99) is 1.9 times of the *a*_*2*_ slope (378.97). According to theoretical calculation, the binding energy of CDHis dimer is −25.6 kcal/mol, demonstrating the spontaneous formation of dimers. Thus, the formation process of CDHis dimers can explain the two stages in Fig. 4b. The increase of FL intensity involves two processes: the dimer formation and increase in dimer amount, whereas the increase of dimer amount is dominant once over the critical concentration (~2.5 × 10^−3^ M) as the dimers have already been well formed. To conform the formation of CDHis dimer, CDHis solution in higher concentration was tested by the gel permeation chromatography (GPC) (Supplementary Fig. 19). The calculated molecular weight is around 3375 g/mol, which matches with the molecular weight of CDHis dimer. The concentration-dependent FL spectra of CDTrp solution and the plots of FL intensity vs. CDTrp concentration were also obtained (Supplementary Fig. 20). It is shown that the FL intensity of CDTrp levels off and even slightly decreases when the CDTrp concentration keeps increasing. The increasing concentration of CDTrp enhances the self-absorption and exciton-exciton interaction, inducing a predominant quenching effect over the enhancing effect, thus weakening the luminescence^29^. According to theoretical calculation, the binding energy of CDTrp dimer is −30.3 kcal/mol, demonstrating the spontaneous formation of CDTrp dimers. The conformation of CDTrp dimer is shown in Supplementary Fig. 21. As shown in Fig. 4c, d, the abundant hydrogen bonds in the intra- and intermolecular CDHis dimer structure facilitate the conjugation of clustered chromophores; meanwhile the intermolecular O···O and N···O interactions in CDHis dimers further strengthen the nonconventional clusteroluminescence. To sum up, the conformation rigidification through abundant hydrogen bonds and the effective intramolecular and intermolecular through-space conjugation in CDHis lead to the unconventional bright emission.

By comparing the emission spectra of CDHis with different concentrations from 0.1 to 5 mM (Supplementary Fig. 22), the slight red-shifts of the emission peak with the increase of CDHis concentration are observed, which suggests the formation of excimers at higher concentration^51^. The excitation spectra for all the emission bands at different concentrations of CDHis are provided. As shown in Supplementary Fig. 23, when fixing the emission wavelength at 470 nm, the excitation peaks of CDHis at 290 nm and 390 nm show obvious red-shift with the increase of CDHis concentration. This result suggests the presence of static excimers at higher concentrations. The emission-wavelength-dependent excitations for both excitation peaks at 290 nm and 390 nm at low concentration from 0.2 to 0.5 mM are observed. However, at high concentration from 1 to 5 mM, the red shift of 390 nm peak is observed with the increase of the emission wavelength, while the position of 290 nm peak does not change with the increase of the emission wavelength. These results indicate the heterogeneity in the excited states in CDHis at different concentrations.

**Fig. 5:**
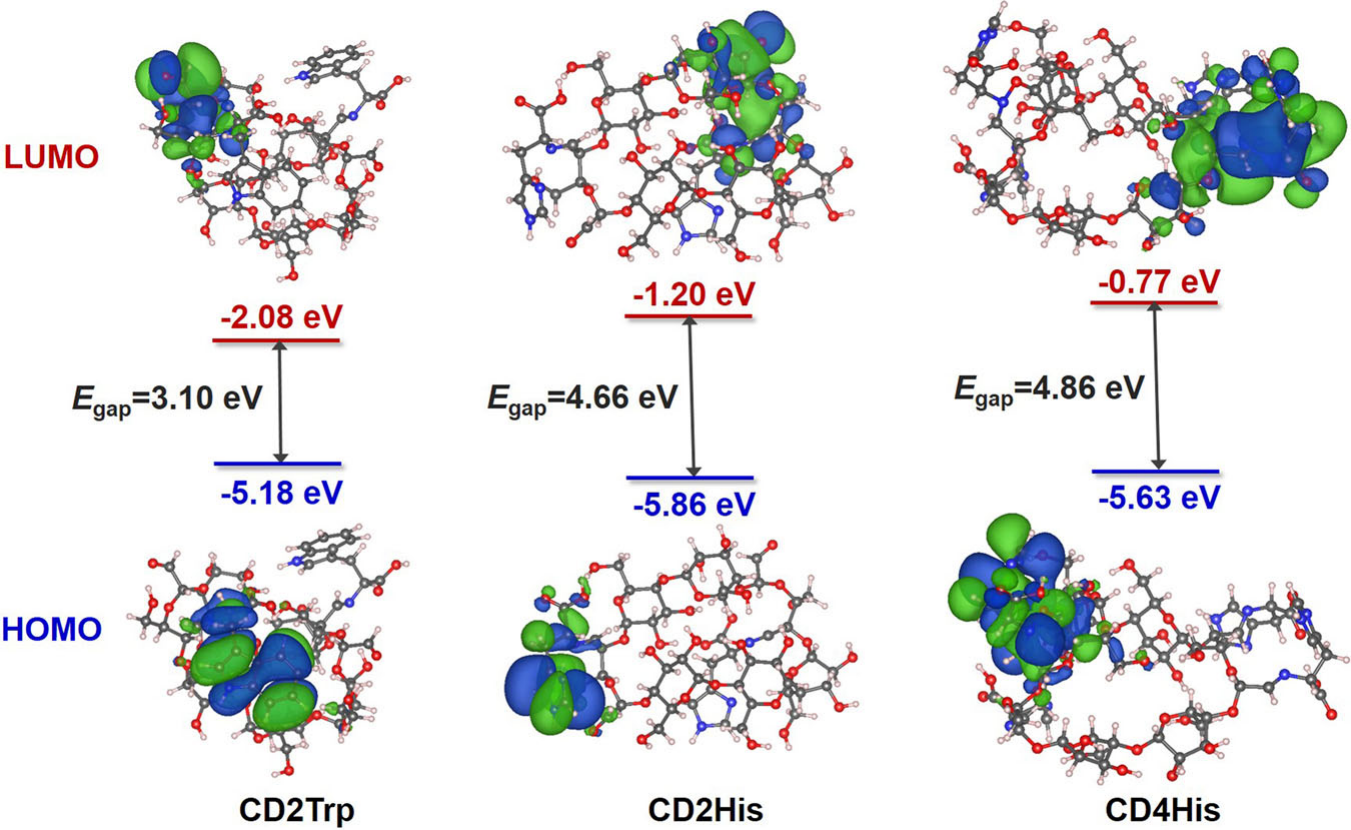
Electron density distributions of HOMO and LUMO levels of CD2Trp, CD2His, and CD4His monomers at B3LYP/6-311G(d) level. The 2, 2, and 4 in CD2Trp, CD2His, and CD4His represent the stoichiometric ratios of AAs to CDA. HOMO the highest occupied molecular orbital, LUMO the lowest unoccupied molecular orbital, and Egap the energy gap between LOMO and HUMO.

To gain insight into the nature of the ground electronic states of CDAA molecules, theoretical calculations on their electronic structures and energy levels were performed using the density functional theory (DFT) method at the B3LYP/6-311 G(d) level. Using CD2Trp, CD2His, and CD4His as the representative CDAA with different types and amounts of conjuncted AAs, their optimized conformations and molecular orbital surfaces of HOMO and LUMO are illustrated in Fig. 5. All the HOMO orbitals locate on the grafted amino acid moieties in CDTrp and CDHis molecules. While, the LUMO orbitals locate on adjacent glucopyranoside units or another amino acid moieties. The electron density distribution of HOMO and LUMO levels of all other CDAAs are shown in Supplementary Fig. 24. The separation of the LUMO and HOMO allows excitation of the electrons and charge transfer between these two energy states, leading to the generation of fluorescence^19^. The short-wavelength emission of CDTrp and CDHis locates at 478 nm and 442 nm (Fig. 2), respectively, which matches well with the trend of calculated energy gap (*E*_gap_) of CDTrp (3.10 eV) and CDHis (4.86 eV).

The theoretically obtained absorption spectra and TDDFT computed excited state data of CDTrp and CDHis are presented in Supplementary Figs. 25–27 and Supplementary Tables 1 and 2. The theoretically obtained spectra show absorption bands beyond ~200 nm, including 384.38 nm band (excited energy of 3.225 eV) and 298.07 nm band (excited energy of 4.160 eV) for CDTrp, and 283.39 nm band (excited energy 4.375 eV) and 276.16 nm band (excited energy 4.490 eV) for CDHis. As discussed, the longer excitation bands also exist, but the corresponding transition is forbidden, so the longer absorption bands were not shown in the experimental absorption spectra due to the low-sensitivity absorption characterization. According to the orbitals in the excited state processes, it can be concluded that the local excitation (LE) is primary for CDTrp but the local excitation (LE) and charge-transfer excitation (CT) both exist for CDHis.

### Fluorescent sensing for CTC

**Fig. 6:**
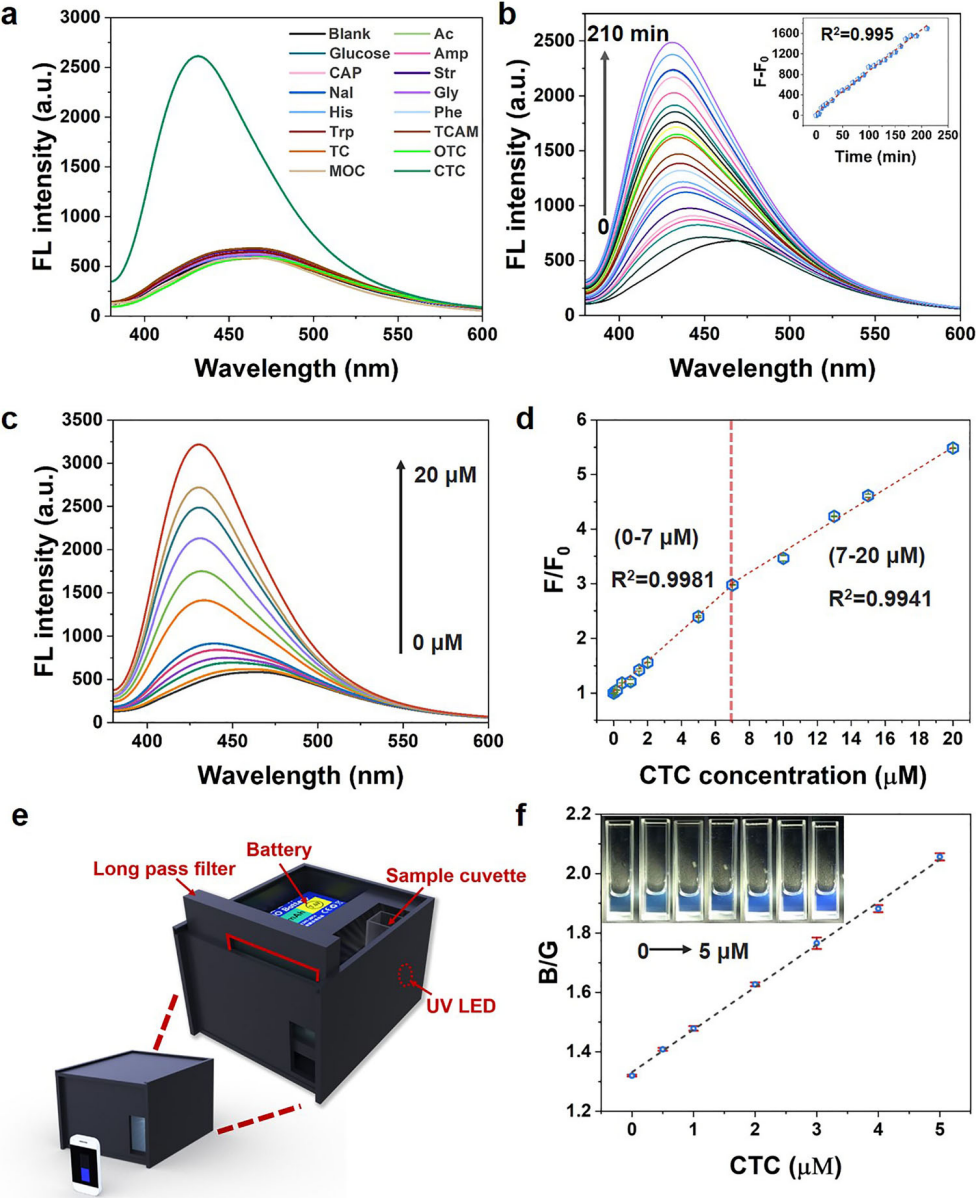
Detection of CTC with CDHis. **a** FL spectra of CDHis after addition of various common organics (λ_ex_ = 356 nm, AC acetic acid, glucose, Amp ampicillin, CAP chloramphenicol, Str streptomycin, Nal nalidixic acid, Gly glycine, His histidine, Phe phenylalanine, Trp tryptophan, TCAM trichloroacetamide, TC tetracycline, OTC oxytetracycline, MOC minocycline). **b** Fluorescence spectra of CDHis (λ_ex_ = 356 nm, 1.25 mM) at different time durations after adding 10 μM CTC. **c** The evolution of fluorescent spectra of CDHis (λ_ex_ = 356 nm) with different CTC concentrations after 120 min interaction. **d** Quantitative change of *F*/*F*_0_ with CTC concentration. **e** smartphone-enabled device for scanometric monitoring of CTC. **f** Plots of B/G values against CTC concentration. B and G values are blue and green color intensities directly scanned from the color scanning app. The error bars were obtained by three parallel experiments. The inset shows the images of CDHis solution (100 μM) with various concentrations of CTC (up to 5.0 μM).

The proof-of-concept of enhanced molecule-level CTE in confined space and the FL mechanism were demonstrated. The nonconventional luminogens involving the through-space conjugation could be tuned with enlarged conjugated groups by introducing foreign chemical. Tetracyclines antibiotics (TCs), as a broad class of veterinary drugs, is ranked second in production and consumption worldwide^52,53^. They are persistent in aquatic environment due to the highly hydrophilic character and low volatility. Among TCs, chlortetracycline (CTC) is the most used antibiotic in swine production in USA and has a long half-life in animal tissues^54,55^. Therefore, it is highly desirable to detect TCs, especially CTC in water body. In recent studies, most fluorescence sensors, e.g., MOFs^56^ and carbon dots^57^ based sensors, cannot discriminate CTC from other TCs. Although some sensors, e.g., CuInS_2_/ZnS quantum dots^58^ and Au nanoclusters^59^, are reported for selective detection of CTC, the sensitivity of these sensors still needs improvement (Supplementary Table 3). Thus, highly selective and sensitive fluorescence sensor is needed for CTC detection. TCs have a strong tendency to form complexes owing to the multiple O- and N-containing moieties^60,61^. Figure 6a shows the effect of TCs including chlorotetracycline (CTC), tetracycline (TC), oxytetracycline (OTC), minocycline (MOC) and other common small organics on the FL intensity of CDHis. Among these organics, only the addition of CTC leads to a significant change (blue-shift emission) to the fluorescent emission of CDHis. In contrast, there was no change in fluorescence after the addition of TC, OTC, and MOC with similar molecular structures. This result indicates the specific molecular recognition between CDHis and CTC. The dynamic fluorescent responses of CDHis to CTC are shown in Fig. 6b, which present blue-shift emissions (Supplementary Fig. 28) and enhanced FL intensity with the increase of interaction time. The change of fluorescence intensity (F-F_0_) shows a perfect linear correlation with the interaction time (*R*^2^ = 0.995).

To determine the feasibility and sensitivity of CTC detection, different concentrations of CTC (0–20 μM) were tested under the excitation at 356 nm after 120 min interaction. As shown in Fig. 6c, the fluorescence intensity increases and the peak blue shifts from 465 nm to 430 nm with the increase of CTC concentration. The highest fluorescence (*F*/*F*_0_) shows perfect linear correlation with CTC concentration in the range of 0–7 μM (*R*^2^ = 0.9981) and 7–20 μM (*R*^2^ = 0.9941), respectively (Fig. 6d). The lower limit of detection (LOD, S/N = 3) was calculated as 0.012 μM, which is quite competitive compared with other similar fluorescence materials for CTC detection, as summarized in Supplementary Table 3. The above results confirm the high selectivity and sensitivity of CDHis for CTC detection. Finally, we designed and constructed a smartphone-enabled device for scanometric monitoring of CTC (Fig. 6e). The fluorescence changes of CDHis enable the real-time/on-site visual detection of antibiotic by using a smartphone with easy-to-access color scanning application (App). The blue and green color intensities (B and G values) of the fluorescent images can be directly scanned from the App (Fig. 6f). Based on the ratio of B/G value and CTC concentration, calibration curves can be set up and utilized for quantitative detection of CTC. The determination of CTC with the homemade smartphone-enabled device can be accomplished within 30 min, which indicates the significant promise of CDAAs as fluorescence probe in trace chemical analysis in aqueous solution.

### Sensing mechanism

**Fig. 7:**
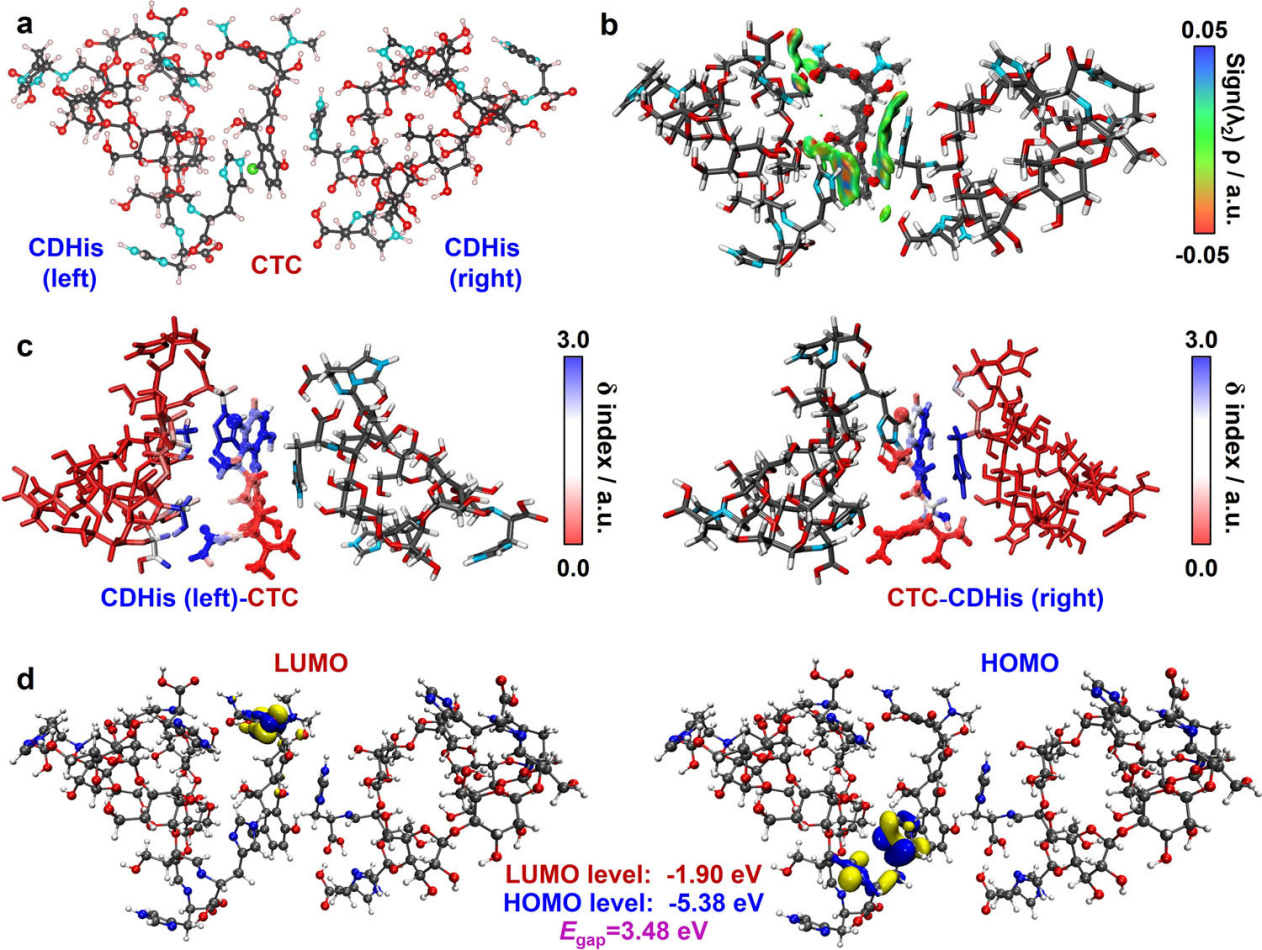
Binding geometry of CDHis and CTC. **a** The optimized binding geometry of the CDHis•CTC•CDHis complex at the B3LYP-D3/6–311 G(d)/SMD(water) level of theory. **b** Independent gradient model (IGM) analysis of CDHis•CTC•CDHis complex. δg^inter^ = 0.01 a.u. isosurfaces colored by the sign of (λ_2_)ρ for the CDHis and CTC complex. The red indicates strong attraction, while the blue indicates strong repulsion. **c** The atoms in CDHis (left), CTC and CDHis (right) molecules are marked to show their contributions to the complexation. The red indicates low contribution to the complexation, and the blue indicates the high contribution. **d** Theoretical calculations of orbital surfaces of HOMOs and LUMOs based on the CDHis•CTC•CDHis complex at B3LYP/6-311 G(d) level.

To elucidate the molecular recognition mechanism of CDHis for CTC, the luminescence lifetimes of CDHis and CDHis•CTC complex were recorded (Supplementary Fig. 29). The average lifetime (τ_av_) of CDHis and CDHis with the addition of CTC was determined as 6.11 ns and 6.26 ns, respectively. The highly similar lifetimes indicate the predominant interaction in the ground state due to the formation of CDHis•CTC complex. Besides, the excitation wavelength of CDHis•CTC complex also blue shifts to 356 nm (Supplementary Fig. 30). These results indicate that CTC has obvious effect on the electron transition in CDHis molecule. To further understand the molecular recognition behavior between CDHis and CTC, the optimized binding geometry was theoretically calculated and independent gradient model (IGM) analysis was conducted. CDHis is electron-rich, especially at its rim with His moiety, and CTC is electron-deficient with multiple O- and N-containing groups. CDHis and CTC molecules tend to approach each other in a complementary manner as shown in the optimized binding geometry in Fig. 7a. Compared with CDHis∙CTC complex (ratio of CDHis:CTC is 1:1, Supplementary Figs. 31 and 32) with a binding energy of −24.90 kcal/mol, the CDHis•CTC•CDHis complex (ratio of CDHis:CTC is 2:1) is more likely to happen with lower binding energy of −63.97 kcal/mol.

Moreover, the IGM analysis^62^ (Fig. 7b) reveals the strong N–H···O hydrogen bonds between amide group in CTC and carbonyl group of His moiety in CDHis and the O–H···O hydrogen bonds between hydroxyl group in CTC and glucopyranoside units in CDHis (red areas in the isosurfaces). The green areas in the isosurfaces indicate the existence of (1) C–H···O weak hydrogen bonds and (2) π–π stacking interactions of the imidazole rings in His moiety and aromatic ring in CTC. The marked atoms of CDHis and CTC according to their contributions to the molecular recognition clearly shows that the main contribution derives from the imidazole rings of His moiety in CDHis and aromatic rings in CTC, though the glucopyranoside units in cyclodextrin rim also provide weak interactions (Fig. 7c). As a result, the synergistic effect of these different interactions contributes to the strong and specific binding between CDHis and CTC.

We further verified the complexation by UV absorption spectra and circular dichroism spectra of the CDHis and CTC complex system. As shown in UV absorption spectra (Supplementary Fig. 33), the typical peak from CTC at 275 nm red shift and the peak at 365 nm blue shift after complexing with CDHis. As shown in Supplementary Fig. 34, the obvious blue shifts of the bands for A, B, C and D ring in CTC molecule are observed, which may be caused by the intermolecular hydrogen bonds. The most significant shift occurs in the D and A rings in CTC molecule, which indicates that the main recognition site locates at the D and A rings when interacting with CDHis. This result is consistent with the theoretical calculations. The molecular orbital surfaces of HOMOs and LUMOs of CDHis•CTC•CDHis complex are shown in Fig. 7d. The HOMO orbitals still locate on the His moieties in CDHis molecule, but the LUMO orbitals transfer to the A ring in CTC, which leads to the evolution of emission wavelength. Besides, with the reduction of the energy band gap (*E*_gap_ = 3.48 eV), the complex molecules are easier to be excited and the molecules in the excited state tend to return to the ground state in the form of radiation because of the rigidified conformation, leading to enhanced fluorescence.

## Discussion

We report a new type of clusteroluminogens through modification of cyclodextrin with amino acids. The synthesized nonconventional chromophores show bright fluorescence even in dilute solution. The unconventional fluorescence was attributed to enhanced intermolecular and intramolecular clusterization of chromophores including –C=N, –COOH, and –OH in cyclodextrin-based confined space and simultaneous conformation rigidification originated from hydrogen bonds and strong complexation interactions between the metal cation and carboxylic anions in CDAA molecules. The newly prepared CDHis was demonstrated for molecular recognition of chlortetracycline towards tetracycline antibiotics of similar molecular structures. This work provides a universal strategy to synthesize nonconventional luminogens with bright fluorescence in dilute solution based on molecular-level enhanced clusterization-triggered emission mechanism. The CDAA molecules present promising prospect as fluorescence probe in sensing and imaging applications.

## Methods

### Synthesis of CDAAs

β-cyclodextrin aldehyde (CDA) was first obtained through partial oxidation of β-cyclodextrin^63,64^. Typically, CD (15 g) were dispersed in 100 mL H_2_O, followed by the addition of sodium periodate (6 g). After stirred for 3 hours under dark condition and filtered with 0.22 μm membrane, CDA were obtained by precipitation using anhydrous ethanol (400 mL) and then washed with ethanol/water mixture (80/20, V/V). For the synthesis of CDHis, CDA (0.1 mmol, 0.113 g) and histidine (0.6 mmol, 0.093 g) were dissolved in 20 mL H_2_O. The above solution was stirred at 80 °C at 600 rpm for 2 hr. The resultant CDHis were dialyzed against ultrapure water (MWCO = 1000 Da) for 24 h, and the dialysate was lyophilized and collected for characterization and further use. For CDTrp, CDLys, and CDArg, tryptophan (0.2 mmol, 0.041 g), lysine (0.4 mmol, 0.059 g) and arginine (0.4 mmol, 0.070 g) were added instead of histidine, followed by the same procedure with CDHis. For other CDAAs including CDGly, CDIle, CDMet, CDCys, CDGlu, CDGln, CDAsn, and CDPhe, the synthesis processes were same with that of CDHis except that amino acid including glycine (0.6 mmol, 0.045 g), isoleucine (0.8 mmol, 0.105 g), methionine (0.6 mmol, 0.090 g), cysteine (0.2 mmol, 0.025 g), glutamic acid (0.8 mmol, 0.118 g), glutamine (0.6 mmol, 0.088 g), asparagine (0.6 mmol, 0.080 g), or phenylalanine (0.6 mmol, 0.099 g) was added, respectively, and the pH value of the solution was adjusted to 8-9 with NaOH solution. Other experimental details are shown in the Supplementary Information.

## Supplementary information

Supplementary Information

## Data Availability

The authors declare that the data supporting the findings of this study are available within the paper and its Supplementary Information files. Source data are provided with this paper.

## Source data

Source Data
