# Supplementary material for: Molecular-level enhanced clusterization-triggered emission of nonconventional luminophores in dilute aqueous solution
Source: Nat Commun. 2023 Jan 25;14:409. doi: 10.1038/s41467-023-36115-w (PMC9876902; doi:10.1038/s41467-023-36115-w)
Supplement: Supplementary file 1 — Supplementary Information [file 41467_2023_36115_MOESM1_ESM.pdf]

Supplementary Information

Molecular-level enhanced clusterization-triggered emission of nonconventional luminophores in dilute aqueous solution

Qiuju Li[1], Xingyi Wang[1], Qisu Huang[1], Zhuo Li[1], Ben Zhong Tang[2*], Shun Mao[1*]

[1] *College of Environmental Science and Engineering, State Key Laboratory of Pollution Control and Resource Reuse, Tongji University, 1239 Siping Road, Shanghai 200092, P.R. China*

[2] *School of Science and Engineering, Shenzhen Key Laboratory of Functional Aggregate Materials, The Chinese University of Hong Kong, Shenzhen City, Guangdong 518172, P.R. China*

*E-mail: tangbenz@cuhk.edu.cn (B. Tang), shunmao@tongji.edu.cn (S. Mao)

Experimental Section

1. Characterizations

The photoluminescence spectra were collected on Hitachi Fluorescence Spectrophotometer F-7100 with an excitation source of xenon lamp. The absolute fluorescence quantum yields of CDHis in ultrapure water was measured using Edinburgh FLS1000 fluorescence spectrophotometer equipped with an integrating sphere. The UV-Vis absorption spectra were recorded on UV-Vis spectrophotometer TU-19 (PERSEE). Fourier transform infrared (FTIR) spectra were collected with a Perkin-Elmer Spectrum GX. The X-ray photoelectron spectroscopy (XPS) spectra were conducted with Thermo K-alpha instrument. X-ray diffraction (XRD) patterns were collected on Bruker D8 Advance X-ray diffractometer. The Circular dichroism spectra were measured on Chirascan CD spectrometer (Jasco-815). The high-resolution electrospray ionization-mass spectrometry (HRMS) of CDAAs were collected on Thermo Scientific Q Exactive using negative ion mode. The main fragmented peaks were analyzed and the detailed mass data are listed below. The NMR spectra were recorded on a Bruker Avance III HD 400 MHz instrument at room temperature.

The full characterization data (^{1}H NMR, ^{13}C NMR, and HRMS) of CDAAs are listed as follows:

CDHis: ^{1}H NMR (D_2O): δ = 8.37, 7.95, 7.69, 7.43, 7.21, 6.98, 6.73, 6.37, 4.70, 3.91, 3.90, 3.89, 3.88, 3.58, 3.17, 3.16, 3.13, 3.12, 3.07, 3.05, 3.03, 3.01 ppm; ^{13}C NMR (400 MHz, solid state): δ = 174.1, 172.0, 135.4 126.4, 114.6, 73.8, 27.4 ppm; HRMS $[M+2H]^-$ calculated for $C_{66}H_{94}O_{39}N_{12}$ (CD4His) = 1681.5; found = 1682.0.

CDAsn: ^{1}H NMR (D_2O) δ=8.32, 6.59, 6.53, 5.28, 4.95, 4.94, 4.70, 4.02, 3.87, 3.84, 3.83, 3.82, 3.81, 3.80, 3.78, 3.75, 3.74, 3.73, 3.72, 3.66, 3.54, 3.53, 3.52, 3.51, 3.48, 3.46, 3.44, 2.94, 2.87, 2.82, 2.81, 2.78, 2.77, 2.71, 2.69, 2.66, 2.64, 2.60, 2.57, 2.54, 2.52, 2.50 ppm; ^{13}C NMR (400 MHz, solid state): δ = 274.9, 175.5, 103.1, 81.8, 78.7, 76.1, 73.2, 60.0, 50.9, 44.1, 34.5 ppm; HRMS $[M-8H]^-$ calculated for $C_{54}H_{84}O_{41}N_6$ (CD3Asn) = 1465.2; found = 1464.5.

CDCys: ^{1}H NMR (D_2O): δ = 7.18, 7.03, 6.53, 5.29, 4.96, 4.95, 4.70, 4.56-4.53, 4.51, 4.23, 4.21, 4.18, 4.07-4.04, 4.02-3.97, 3.96, 3.95, 3.88, 3.86, 3.83, 3.82, 3.78, 3.77, 3.74, 3.59, 3.58, 3.55-3.52, 3.50, 3.47, 3.45, 3.44, 3.43, 3.41, 3.40, 3.39, 3.38, 3.36, 3.29, 3.28, 3.26, 3.25, 3.20, 3.17, 3.16, 3.14, 3.09, 3.07, 3.06, 3.04, 2.73, 2.72, 2.69, 2.61, 2.58, 2.57, 2.55, 2.49, 1.92, 1.79 ppm; ^{13}C NMR (400 MHz, solid state): δ = 174.8, 173.0, 102.9, 81.7, 72.8, 61.7, 52.6, 36.5 ppm; HRMS $[M+O]^-$ calculated

for $C_{51}H_{81}O_{38}N_3S_3$ (CD3Cys) = 1456.4; found = 1456.2.

CDGln: ^{1}H NMR (D_2O): δ = 8.32, 7.52, 6.78, 5.39, 5.28, 4.95, 4.94, 4.70, 4.02, 3.87, 3.84-3.80, 3.78, 3.75-3.72, 3.66, 3.54-3.51, 3.48, 3.46, 3.44, 2.94, 2.87, 2.82, 2.81, 2.78, 2.77, 2.71, 2.69, 2.66, 2.64, 2.60, 2.57, 2.54, 2.52, 2.50-2.47, 2.43, 2.41-2.39, 2.37-2.25, 2.23, 2.20, 2.18, 2.17, 2.14, 2.07, 2.04, 2.02, 2.00, 1.98, 1.96, 1.95, 1.93-1.86 ppm; ^{13}C NMR (400 MHz, solid state): δ = 276.4, 180.2, 177.0, 173.5, 103.2, 81.4, 77.5, 73.1, 58.9, 53.8, 29.0, 26.0 ppm; HRMS [M-CNO]$^-$ calculated for $C_{57}H_{90}O_{41}N_6$ (CD3Gln) = 1473.4; found = 1475.5.

CDGlu: ^{1}H NMR (D_2O): δ = 6.40, 5.27, 4.95, 4.94, 4.70, 4.67, 4.58, 3.86, 3.84, 3.81, 3.80, 3.78, 3.75, 3.72, 3.59-3.56, 3.54, 3.53, 3.51, 3.50, 3.48, 3.46, 3.43, 2.59, 2.36, 2.26, 2.24, 2.22, 2.20, 2.18, 2.16, 2.14, 2.02-1.92, 1.90, 1.88, 1.86, 1.84 ppm; ^{13}C NMR (400 MHz, solid state): δ = 181.4, 176.3, 102.9, 81.7, 72.7, 61.5, 55.1, 34.5, 28.7 ppm; HRMS [M-H]$^-$ calculated for $C_{62}H_{94}O_{47}N_4$ (CD4Glu) = 1646.4; found = 1646.5.

CDGly: ^{1}H NMR (D_2O): δ = 6.14, 5.28, 4.95, 4.94, 4.70, 4.66, 4.61, 4.57, 3.87, 3.84, 3.82, 3.80, 3.78, 3.75, 3.74, 3.73, 3.72, 3.58, 3.54, 3.53, 3.52, 3.51, 3.48, 3.46, 3.44, 3.40, 3.22, 3.12, 2.59 ppm; ^{13}C NMR (400 MHz, solid state): δ = 174.3, 103.5, 82.9, 81.1, 79.0, 74.9, 73.4, 42.4 ppm; HRMS [M-5H]$^-$ calculated for $C_{50}H_{78}O_{39}N_4$ (CD4Gly) = 1354.1; found = 1354.3.

CDIle: ^{1}H NMR (D_2O): δ = 6.17, 5.28, 4.95, 4.94, 4.70, 3.87, 3.84, 3.82, 3.81, 3.79, 3.76, 3.74, 3.73, 3.54-3.49, 3.46, 3.44, 1.88, 1.86-1.78, 1.35, 1.34, 1.32-1.29, 1.18, 1.16, 1.14-1.10, 1.08, 1.07, 1.04, 1.02, 0.98, 0.96, 0.91, 0.88, 0.87, 0.84, 0.82, 0.81, 0.79, 0.72, 0.71, 0.65 ppm; ^{13}C NMR (400 MHz, solid state): δ = 175.5, 76.1, 61.5, 36.8, 26.1, 16.4, 13.8, 11.7 ppm; HRMS [M-N]$^-$ calculated for $C_{66}H_{110}O_{39}N_4$ (CD4Ile) = 1569.6; found = 1572.0.

CDLys: ^{1}H NMR (D_2O): δ = 8.31, 7.89, 6.18, 5.27, 4.95, 4.94, 4.88, 4.70, 3.87, 3.84, 3.82, 3.81, 3.79, 3.76, 3.74, 3.73, 3.54-3.49, 3.46, 3.44, 1.88, 1.86-1.78, 1.39-1.29, 1.18, 1.16, 1.14-1.10, 1.08, 1.07, 1.04, 1.02, 0.98, 0.96, 0.91, 0.88, 0.87, 0.84, 0.82, 0.81, 0.79 ppm; ^{13}C NMR (400 MHz, solid state): δ = 181.6, 177.4, 175.1, 103.2, 82.0, 73.0, 59.5, 55.6, 40.6, 32.5, 28.6, 26.1 ppm; HRMS [M+H]$^-$ calculated for $C_{60}H_{102}O_{38}N_6$ (CD3Lys) = 1521.5; found = 1521.9.

CDMet: ^{1}H NMR (D_2O): δ = 6.31, 5.27, 4.94, 4.70, 3.85, 3.83, 3.81, 3.80, 3.78, 3.75, 3.73, 3.72, 3.70, 3.69, 3.68, 3.53, 3.51, 3.50, 3.48, 3.45, 3.43, 3.21, 2.96, 2.88, 2.69, 2.67, 2.65, 2.61, 2.54, 2.52, 2.50, 2.48, 2.47, 2.45, 2.34, 2.32, 2.31, 2.17, 2.16, 2.14, 2.10, 2.09, 2.08, 2.06-2.01, 1.99, 1.98, 1.96,

1.94, 1.92, 1.89, 1.82, 1.77 ppm; ^{13}C NMR (400 MHz, solid state): δ = 177.5, 177.0, 102.6, 83.0, 80.5, 77.6, 74.3, 72.9, 72.1, 60.6, 55.7, 53.7, 32.9, 31.4, 17.1, 15.9 ppm; HRMS $[M-CN]^-$ calculated for $C_{62}H_{102}O_{39}N_4S_4$ (CD4Met) = 1629.7; found = 1629.6.

CDPhe: ^1H NMR (D_2O): δ = 7.49, 7.38, 7.31-7.18, 7.10, 7.09, 7.00, 6.98, 4.94, 4.70, 4.65, 4.01, 3.85-3.80, 3.74, 3.65, 3.53, 3.50, 3.46, 3.40, 3.29, 3.17, 3.15, 3.13, 3.12, 3.00, 2.99, 2.97, 2.95 ppm; ^{13}C NMR (400 MHz, solid state): δ = 175.7, 173.1, 135.9, 130.8, 128.9, 58.9, 57.4, 40.8, 38.1, 31.4, 29.2 ppm; HRMS $[M+5H]^-$ calculated for $C_{78}H_{102}O_{39}N_4$ (CD4Phe) = 1724.7; found = 1724.7.

CDArg: ^1H NMR (D_2O): δ = 8.32, 6.32, 5.27, 5.16, 4.98, 4.95, 4.94, 4.70, 4.60, 4.57, 4.36, 4.31, 4.11, 3.98, 3.85, 3.83, 3.81, 3.80, 3.78, 3.75, 3.73, 3.72, 3.66, 3.62, 3.54-3.51, 3.48, 3.46, 3.44, 3.40, 3.26, 3.24, 3.23, 3.09, 3.07, 3.06, 2.91, 2.90, 2.88, 2.82, 2.59, 2.30, 2.11, 2.09, 1.78, 1.70, 1.67, 1.65-1.60, 1.58-1.51, 1.49, 1.47-1.43, 1.41, 1.40, 1.35, 1.33, 1.06, 1.05, 1.03, 0.98 ppm; ^{13}C NMR (400 MHz, solid state): δ = 182.4, 181.6, 158.2, 103.7, 84.1, 82.4, 73.5, 56.4, 41.9, 32.5, 27.2, 9.9 ppm; HRMS $[M-5H]^-$ calculated for $C_{66}H_{114}O_{39}N_{16}$ (CD4Arg) = 1750.7; found = 1750.7.

CDTrp: ^1H NMR (D_2O): δ = 8.32, 7.61, 7.59, 7.40, 7.38, 7.17, 7.16, 7.14, 7.12, 7.07, 7.04, 6.97, 6.68, 6.20, 5.26, 4.93, 4.92, 4.87, 4.78, 4.70, 4.62, 4.60, 4.55, 3.88-3.85, 3.80, 3.78, 3.76, 3.72, 3.68, 3.66, 3.63, 3.52-3.49, 3.46, 3.44, 3.41, 3.34, 3.33, 3.30, 3.29, 3.17, 3.15, 3.13, 3.11, 2.67, 2.66, 2.52, 2.30, 2.22, 2.15, 2.11, 2.09, 1.78 ppm; ^{13}C NMR (400 MHz, solid state): δ = 220.1, 175.0, 170.4, 136.2, 127.0, 120.4, 112.1, 108.8, 73.8, 57.0, 27.3 ppm; HRMS $[M-3H]^-$ calculated for $C_{64}H_{86}O_{37}N_4$ (CD2Trp) = 1500.4; found = 1500.6.

2. Materials and chemicals

Chemicals including β-cyclodextrin (98%), sodium periodate (AR) Chlortetracycline (CTC, 99.9%), Oxytetracycline (OTC, 99.9%), Tetracycline (TC, 99.9%), Minocycline (MOC, 99%), glucose (99%) were purchased from Aladdin Chemistry Co., Ltd (Shanghai, China). Common amino acids including glycine (Gly, 99.5%), isoleucine (Ile, 99%), methionine (Met, 99%), cysteine (99.8%), glutamic acid (Glu, 99%), glutamine (Gln, 99%), asparagine (Asn, 99%), lysine (Lys, 99%), arginine (Arg, 99%), phenylalanine (Phe, 99%), histidine (His, 99%), tryptophan (Trp, 99%) were supplied by Shanghai Macklin Biochemical Co., Ltd. All chemicals and regents were used without further purification. Ultrapure water (18.2 MΩ·cm) produced from a Milli-Q system was used for all

experiments.

3. Fluorescence detection of chlortetracycline

In the fluorescence detection experiment, 2.5 mM CDHis (1 mL) as probe was mixed with different concentrations of chlortetracycline solution (1 mL). After incubating for 120 min at room temperature, the fluorescence spectra were collected with excitation at 356 nm. For the selectivity test, possible interference substances were tested including other antibiotics of tetracycline (TC), oxytetracycline (OTC), minocycline (MOC), ampicillin (Amp), chloramphenicol (CAP), streptomycin (Str), nalidixic acid (Nal) and small organics of acetic acid (AC), glucose, glycine, histidine, phenylalanine, tryptophan, trichloroacetamide (TCAM).

The fluorescence time-resolved decay spectra were collected using Edinburgh FLS1000 fluorescence spectrophotometer optically excited with pulsed picosecond 375 nm diode laser and were connected to a time-correlated single photon counting (TCSPC) acquisition module. The monitoring wavelength for CDHis and CDHis-CTC solutions was 475 nm. Ludox was used to calculate instrument response factor (IRF). The data were analyzed with the software of Edinburgh instrument using a multiexponential model for lifetime. The average lifetimes (τ_{av}) were calculated with $\tau_{av}=\Sigma B_i\tau_i^2/\Sigma B_i\tau_i$, where B_i is the preexponential for lifetime τ_i.

4. Simulations

The CDA optimum conformation in H_2O was obtained by ORCA quantum chemistry software (Version 5.0.1). The geometry optimization calculations were conducted through BLYP functional and the def2-SVP basis set. The DFT-D3 dispersion correction with BJ-damping was used to correct the weak interaction to improve the calculation accuracy. The SMD implicit solvation model was applied to account for the solvation effect of water.

The geometry optimization of CDAAs were performed with the semi-empirical xtb program on xtb software (version 6.4.1) by the GFN2 parameter set [1,2]. In order to obtain the stable conformation, a molecular dynamics simulation of 20 ps was performed at 413 K based on the xtb method for each structure. During the relaxation process, every structure was collected every 200 fs, and a total of 100 structures were collected. SHAKE algorithm was applies to constrain X-H bonds. The mass of

hydrogen atoms was set to its original mass. Then, each geometry in the trajectory was extracted and optimized separately, finally the one with the lowest energy was represented. The generalized born surface approximation (GBSA) implicit solvation model was employed with water as the solvent. The configurations of the CDAAs were searched using the Molclus program[3]. Orbital energy level analysis was conducted by Multiwfn software[4]. The visualization of the orbitals was performed using VESTA software.

The excited states calculations were conducted with linear response time-dependent DFT (TDDFT) at the optimized ground state geometry. All calculations are performed with the Gaussian 16 package (Rev. C.01)[5] using the hybrid B3LYP functional[6-7] and the 6-311+G(d) basis set. Grimme's D3BJ dispersion correction[8] was applied to improve calculation accuracy. The SMD implicit solvation model was used to account for the solvation effect of water[9].

The geometry optimization of the cluster between CDHis and CTC was performed with the semi-empirical xtb program by GFN2 parameter set. The vibrational frequencies of the optimized structures were carried out at the same level. The interaction energy of the optimized cluster was calculated at the B3LYP/6-311G(d) level of theory with Grimme's D3 dispersion correction via the Gaussian 16 suite of programs. Independent gradient model (IGM) analysis was achieved using the Multiwfn software. The Visual Molecular Dynamics (VMD) program was applied to obtain the color-filled isosurfaces graphs to visualize the molecular orbitals and molecular graphs.

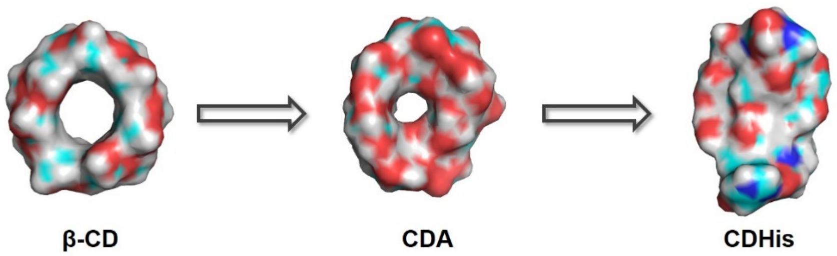

Supplementary Fig. 1. Geometry transformation in the process of CDHis synthesis.

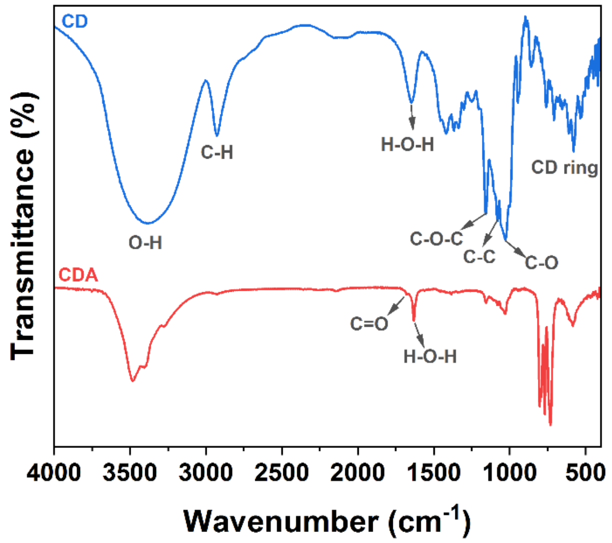

Supplementary Fig. 2. FTIR spectra of pristine CD and CDA.

Based on the FTIR spectra of CDA, the peaks ranging from 950 to 530 cm^{-1} for cyclodextrin ring remains, indicating the complete structure reservation in CDA molecule[10,11]. The typical peaks of C-O-C glucosydic stretching vibration (1158 cm^{-1}), C-C stretching vibration (1084 cm^{-1}), C-O stretching vibration (1028 cm^{-1}) and peaks for CD ring (950-530 cm^{-1}) also remain after the oxidation with $NaIO_4$. Compared with CD, a new peak at 1680 cm^{-1} appears in the FTIR spectra of CDA, indicating the presence of aldehyde groups (-C=O) in the CDA molecule. Moreover, the peak of O–H stretching vibration at 3386 cm^{-1} becomes sharper, mainly due to the cleavage of C-2, C-3-trans-diol position of D-glucose residues and the breakage of the hydrogen network[12]. The peak of C-H almost disappears, which indicates the geometry transformation in CDA molecules.

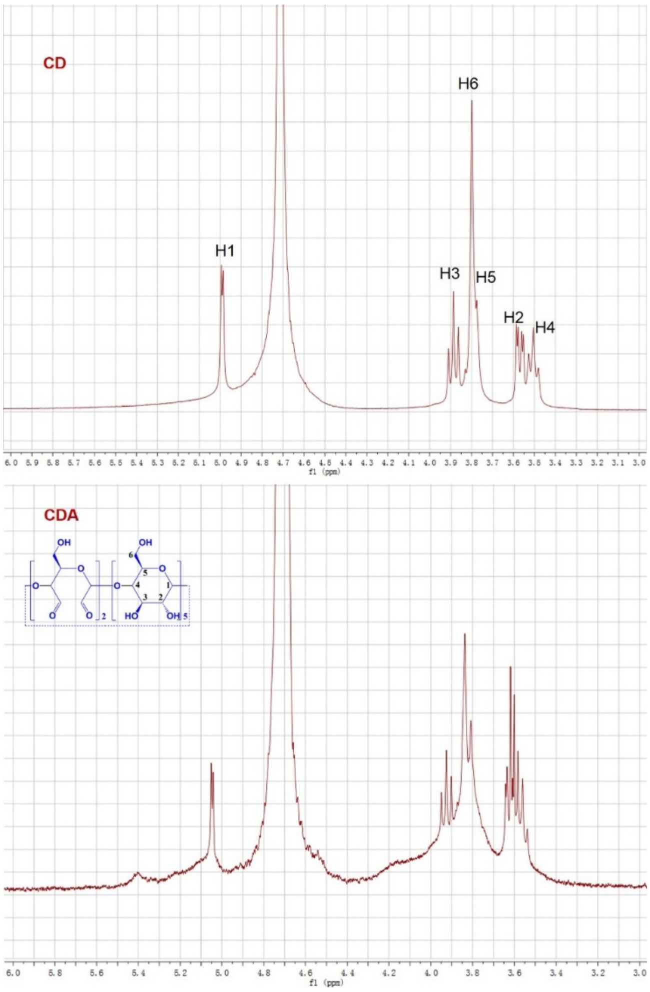

Supplementary Fig. 3. ^{1}H NMR spectra of pristine CD and CDA in D_2O.

The ^{1}H NMR spectra of the pristine CD and CDA samples are shown in Supplementary Fig. 3. The peak intensities for H1-H6 remain unchanged but show slight down-shifts. These results suggest that the cavity structure of CD remains in the oxidized CDA, which is consistent with the results of previous studies[11]. The aldehyde protons peaks are absent in the CDA, probably due to the formation of hemiacetals. Moreover, a new peak is observed at 5.4 ppm, which can be assigned to the protons in −CH groups in the hemiacetals structure in CDA.

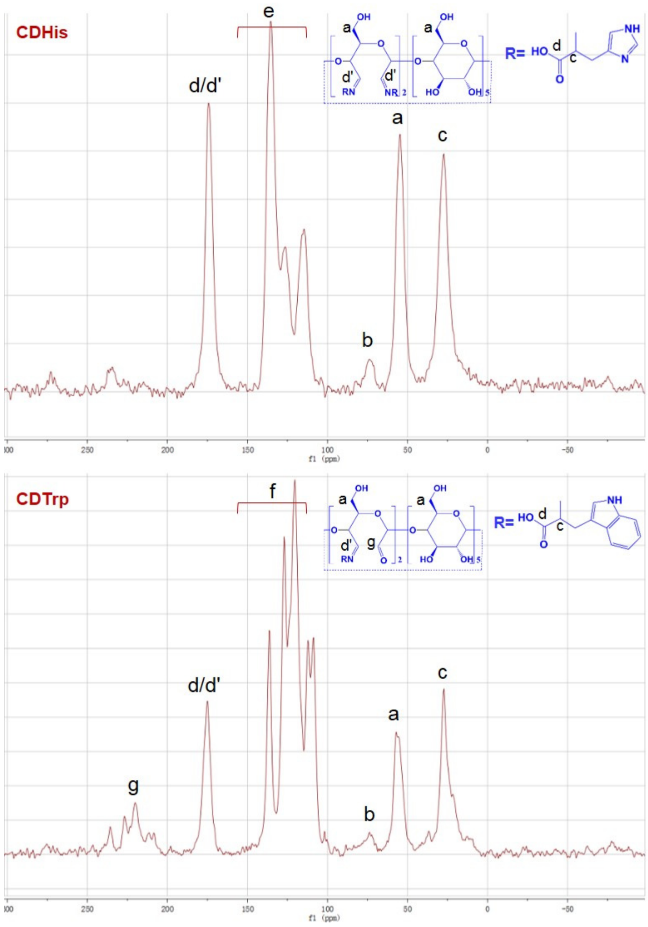

Supplementary Fig. 4. ^{13}C solid-state NMR spectra of CDHis and CDTrp. Resonances labelled by e and f correspond to the grafted iminazole group in histidine moiety and indole group in tryptophane moiety, respectively.

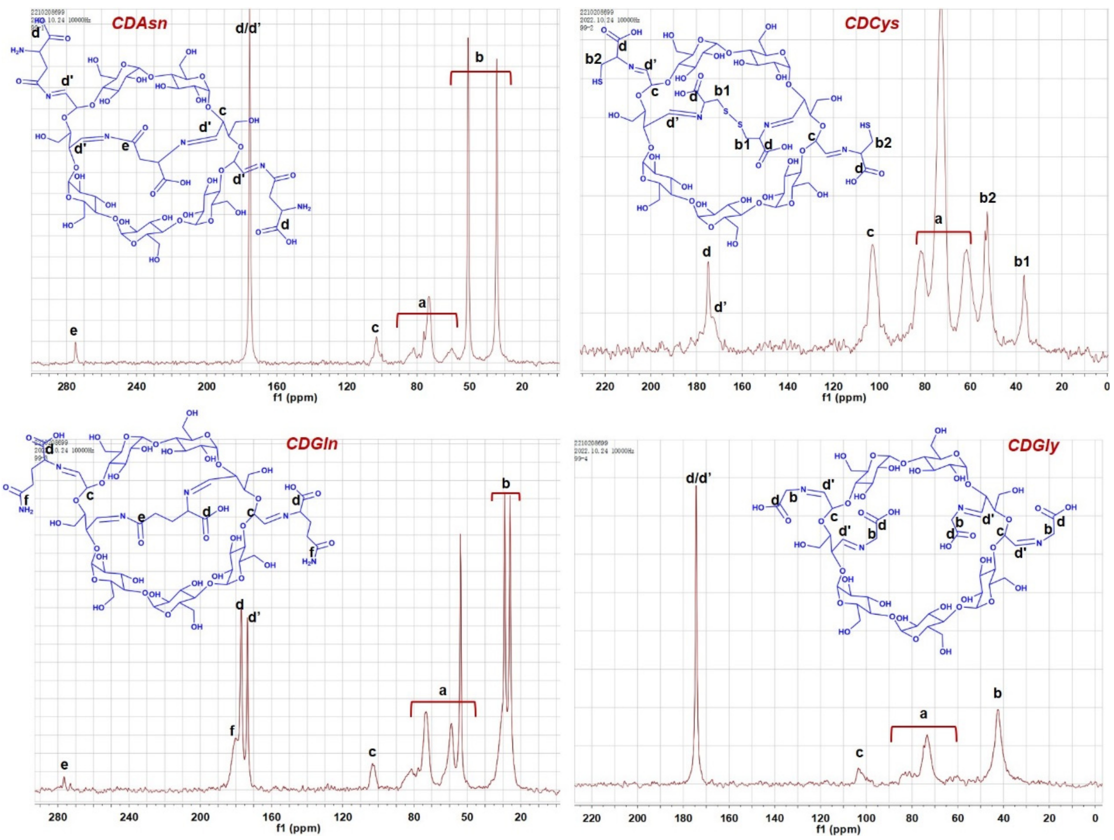

Supplementary Fig. 5. ^{13}C solid-state NMR spectra of CDAsn, CDCys, CDGln, and CDGly. Resonances labelled by a and b correspond to the C signals in glucose moiety in cyclodextrin and in grafted amino acids moiety, respectively.

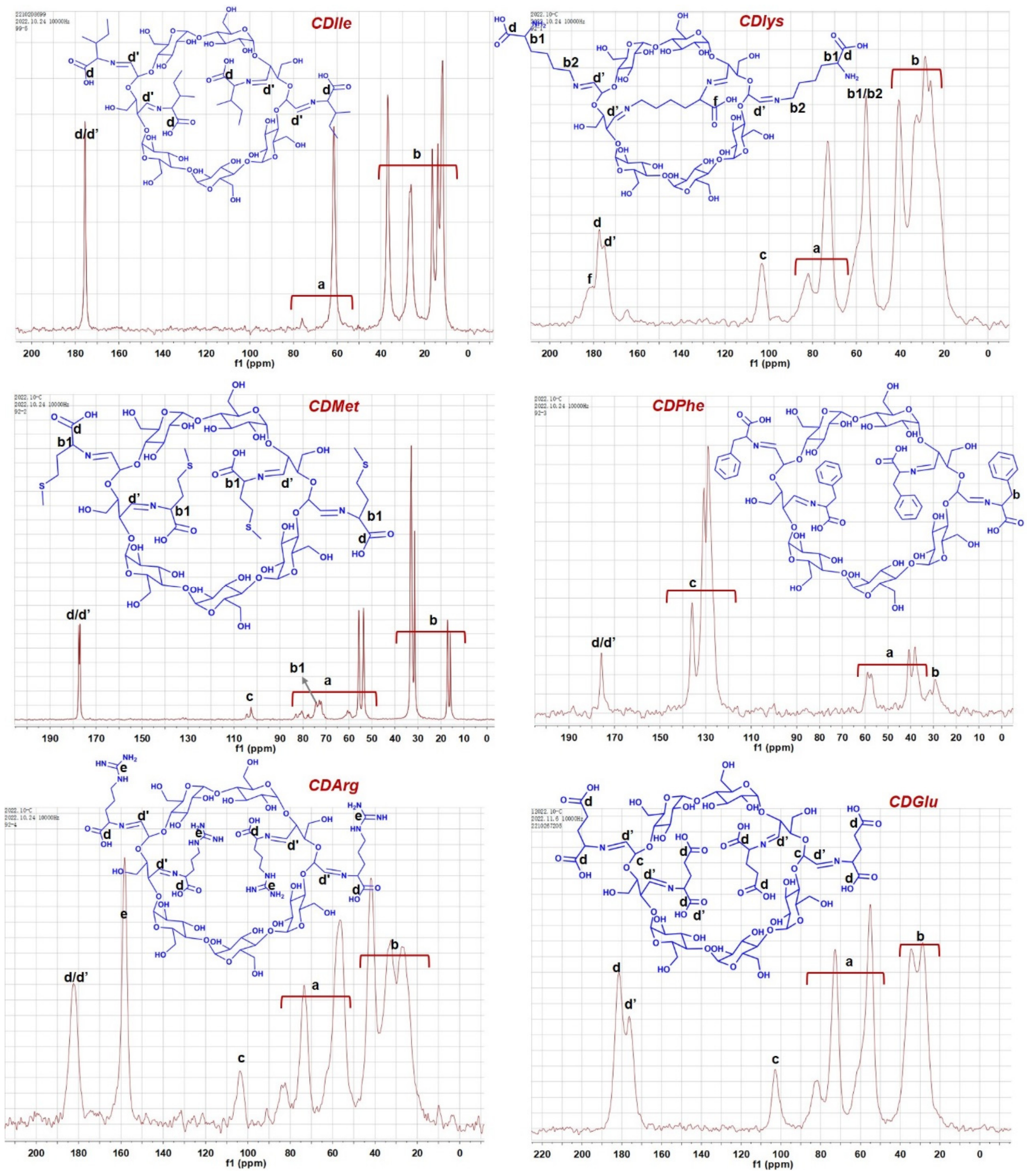

Supplementary Fig. 6. ^{13}C solid-state NMR spectra of CDIle, CDLys, CDMet, CDPhe, CDArg, and CDGlu. Resonances labelled by a and b correspond to the C signals in glucose moiety in cyclodextrin and in grafted amino acids moiety, respectively.

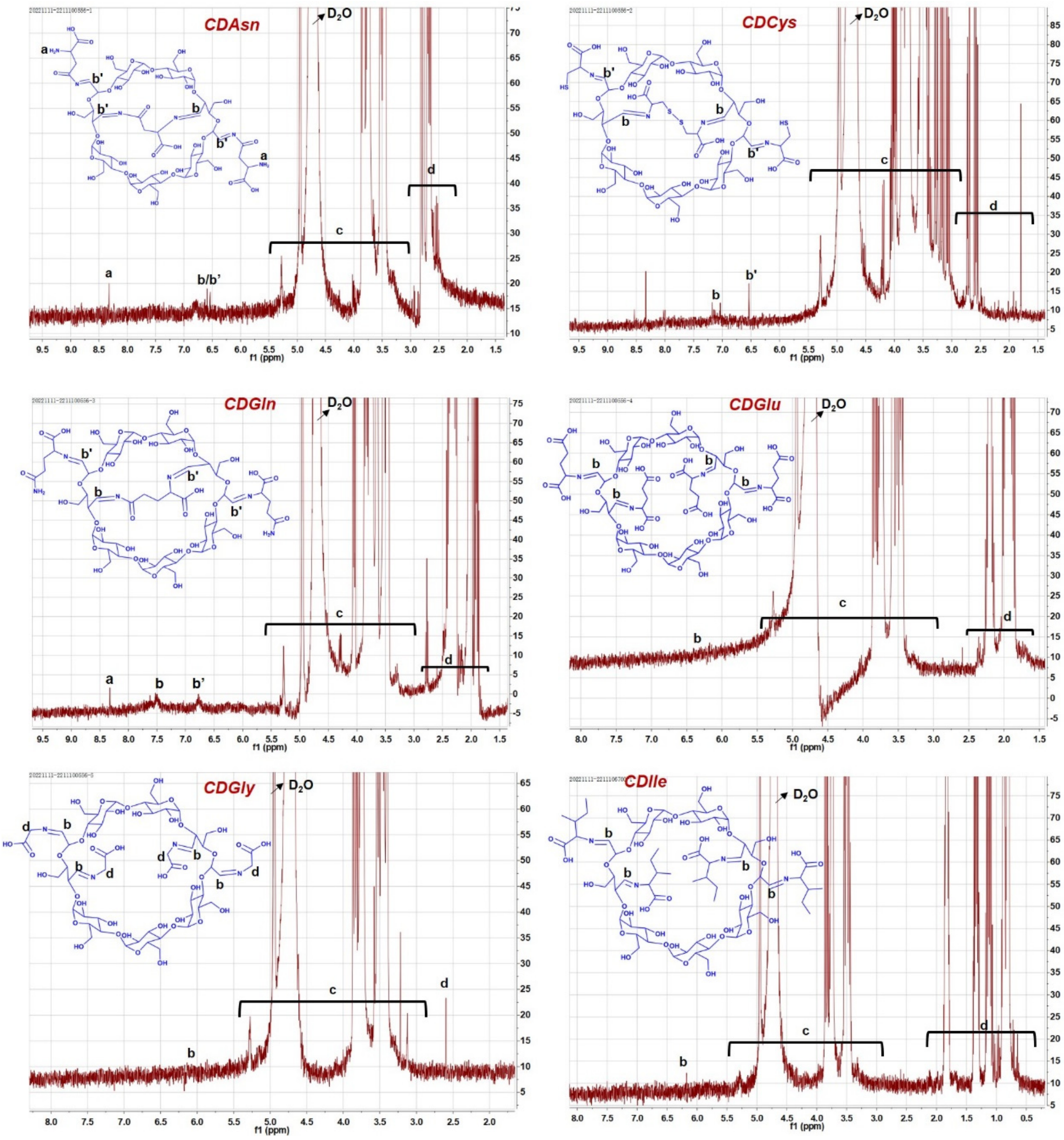

Supplementary Fig. 7. ^{1}H NMR spectra of CDAsn, CDCys, CDGln, CDGlu, CDGly, and CDIle. Resonances labelled by c and d correspond to H signals in the glucose moiety in cyclodextrin and in grafted amino acids moiety, respectively.

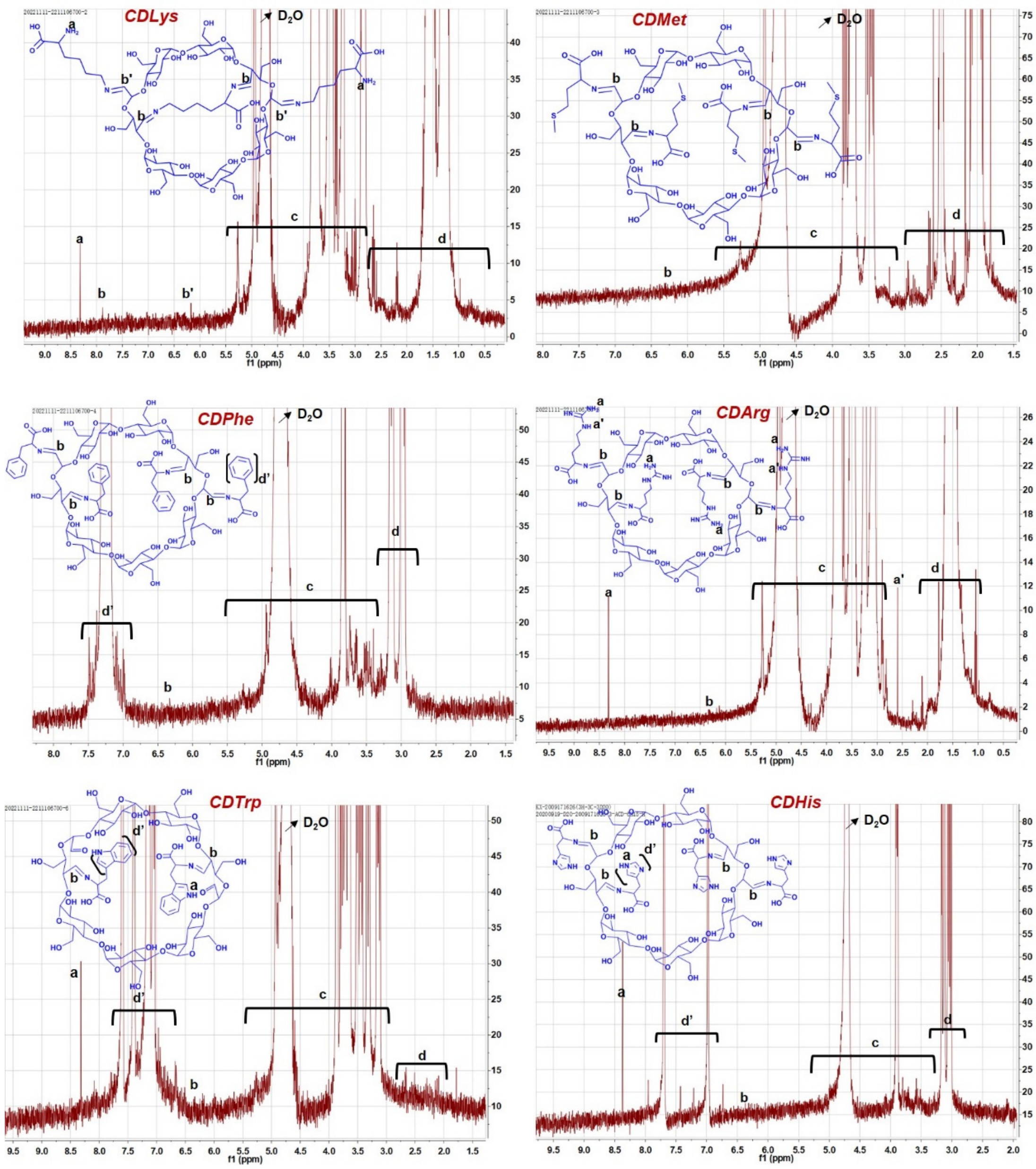

Supplementary Fig. 8. ^{1}H NMR spectra of CDLys, CDMet, CDPhe, CDArg, CDTrp, and CDHis. Resonances labelled by c and d correspond to H signals in the glucose moiety in cyclodextrin and in grafted amino acids moiety, respectively.

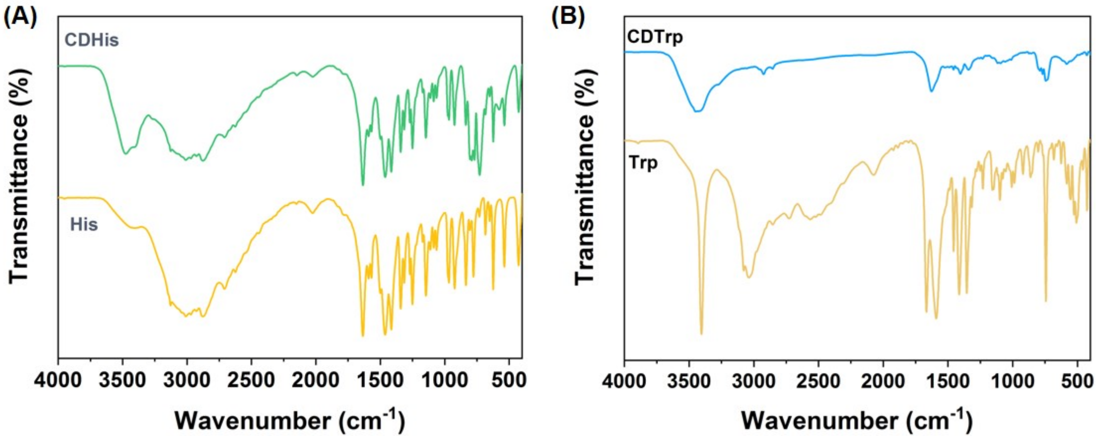

Supplementary Fig. 9. FTIR spectra of (A) His and CDHis and (B) Trp and CDTrp.

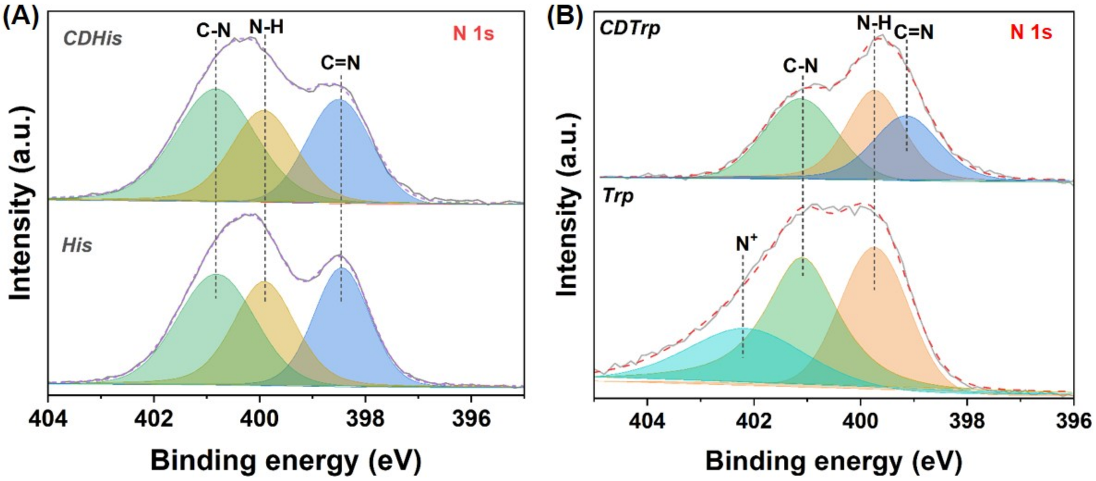

Supplementary Fig. 10. XPS spectra analysis of CDHis and CDTrp. (A) N *1s* XPS spectra of His and CDHis. The ratio of C=N peak area to N-H peak area increase in CDHis compared with that in His. (B) N *1s* XPS spectra of Trp and CDTrp. The C=N peak appears, and the areas of N-H peak and N^+ peak decrease in CDTrp.

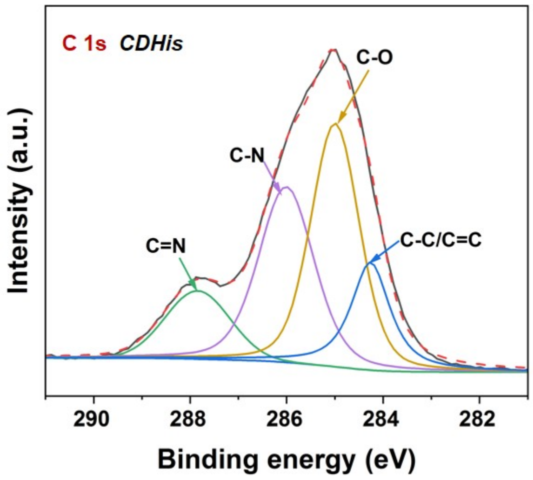

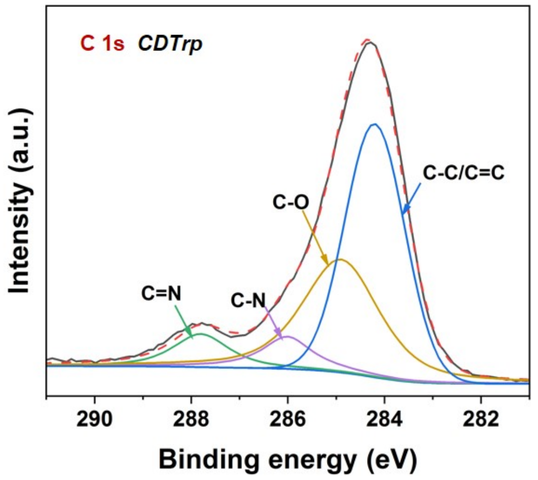

Supplementary Fig. 11. C *1s* XPS spectra of CDHis and CDTrp.

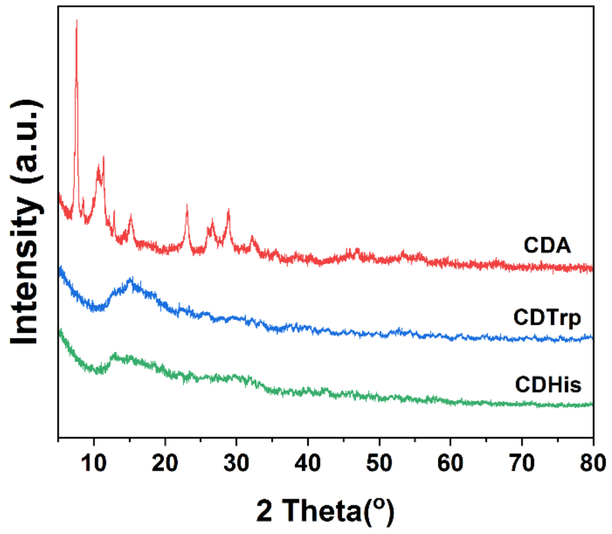

Supplementary Fig. 12. XRD patterns of CDA, CDTrp, and CDHis.

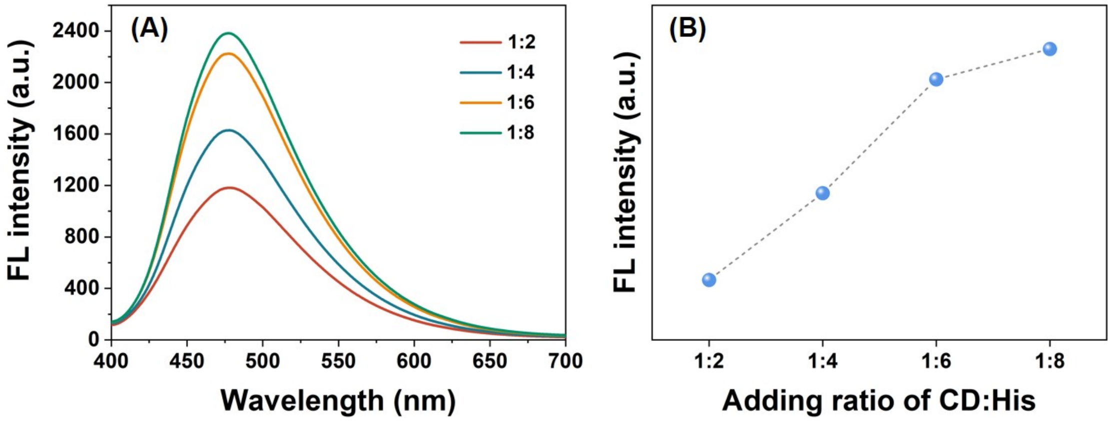

Supplementary Fig. 13. Changes of (A) fluorescence spectra and (B) intensity with different ratios of CD:His. The fluorescence intensity increases with the increase of His component. The optimum adding ratio of CD:His in experiments is set as 1:6 where the amount of His is overused because the theoretical stoichiometric ratio of CD:His is 1:4.

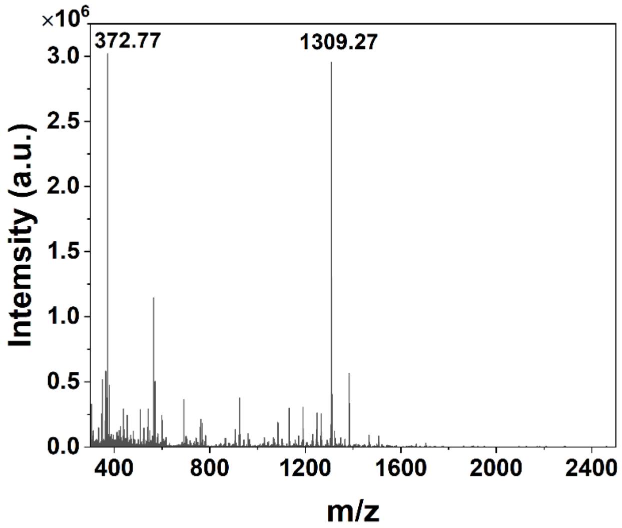

Supplementary Fig. 14. High resolution ESI mass spectrum of CDHis with the peak (*m/z*) at 372.77 and 1309.27, which together matches with the molecular weight of CDHis (1681.5).

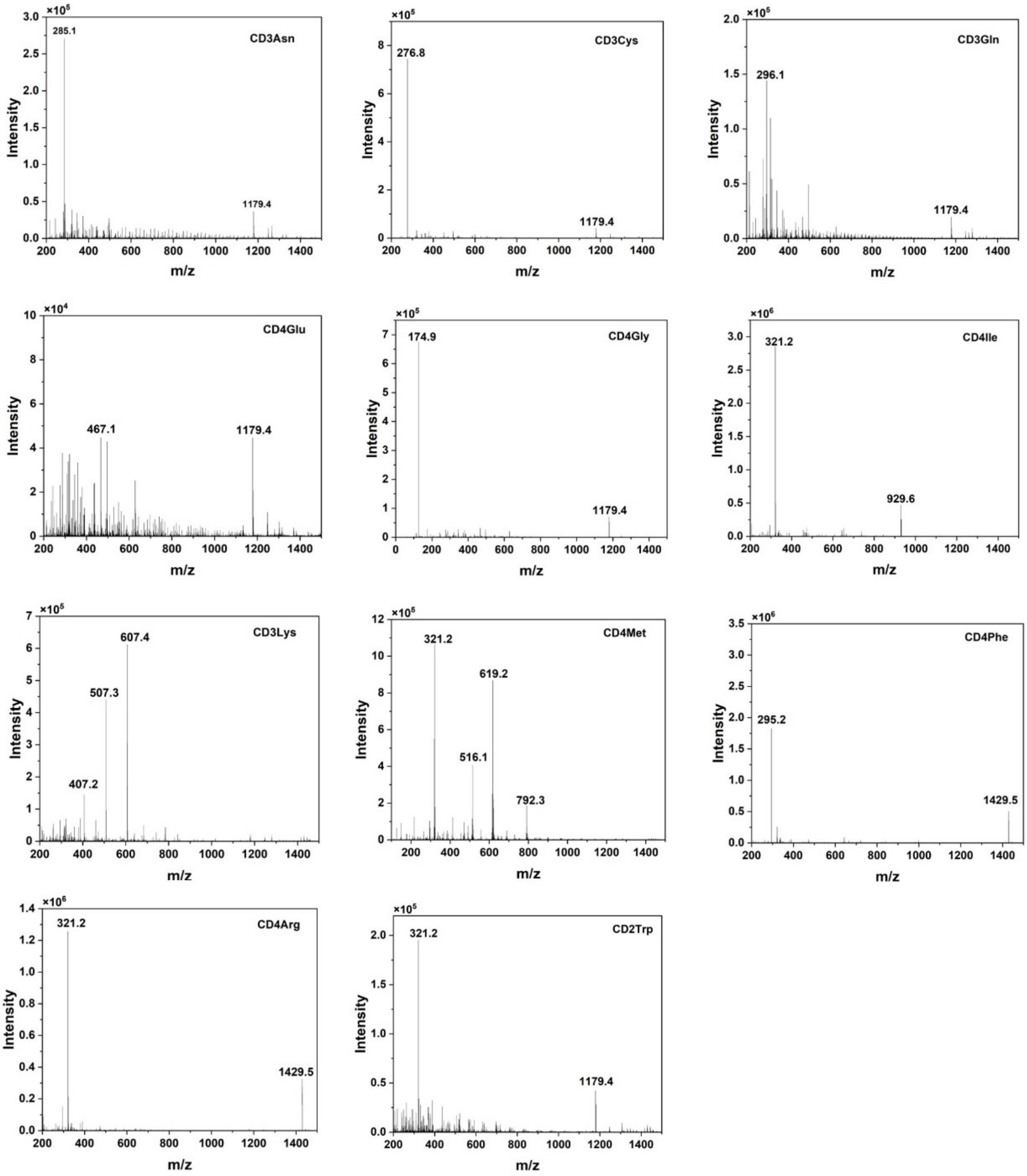

Supplementary Fig. 15. High-resolution ESI mass spectra of other CDAAs including CDAsn, CDCys, CDGln, CDGlu, CDGly, CDIle, CDLys, CDMet, CDPhe, CDArg, and CDTrp. The mass data can interpret the possible number of AAs in CDAAs, from which the stoichiometric ratio of CD:AA is 1:2 in CDTrp, is 1:3 in CDAsn, CDCys, CDGln and CDLys, and is 1:4 in CDGlu, CDGly, CDIle, CDMet, CDPhe, and CDArg.

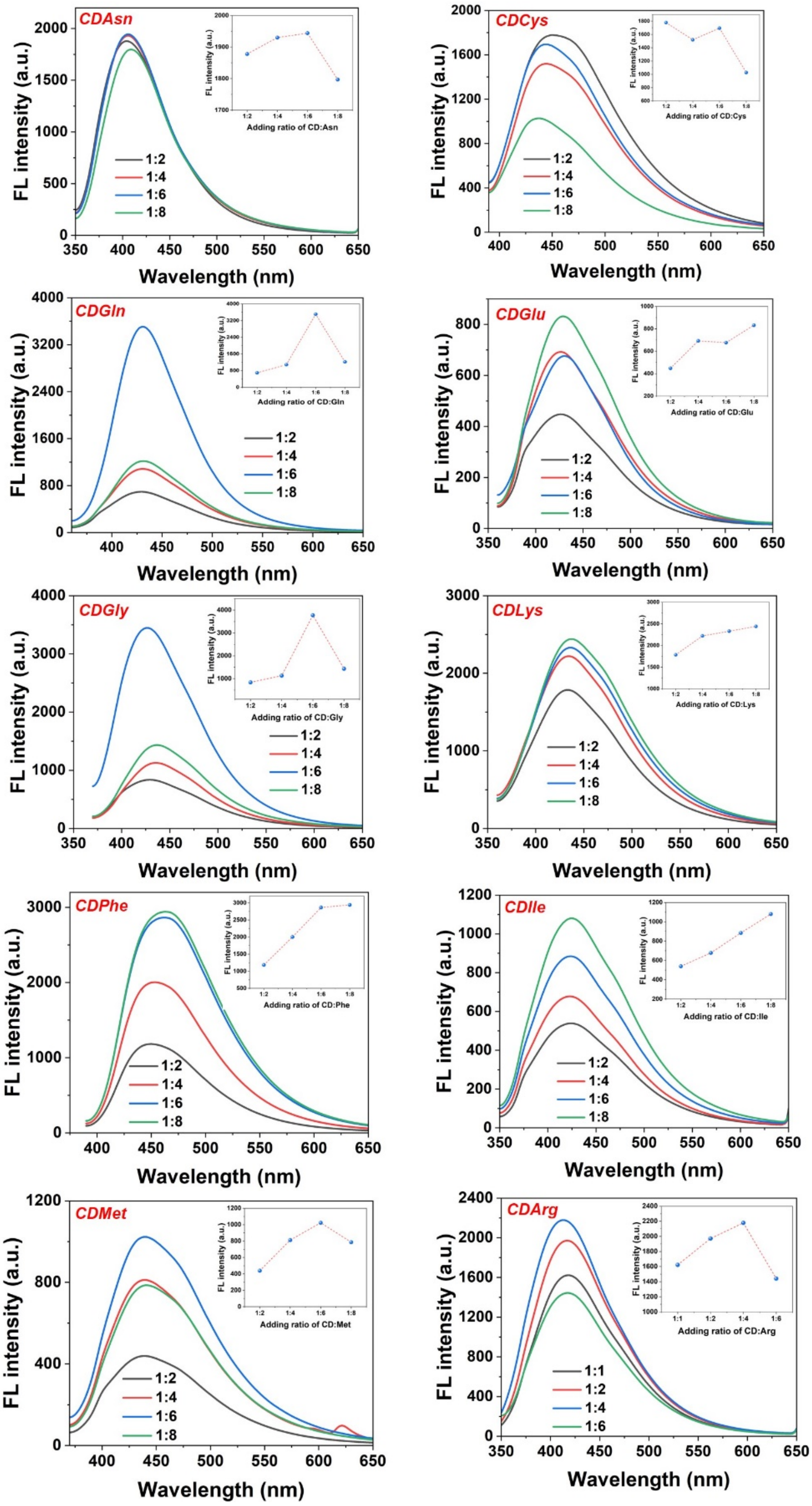

Supplementary Fig. 16. The fluorescence spectra of CDAAs with different AA amounts. The inset shows the fluorescence intensity at different ratios of CD:AA.

Luminophore	Photograph	Quantum yield	Ref.
CDHis	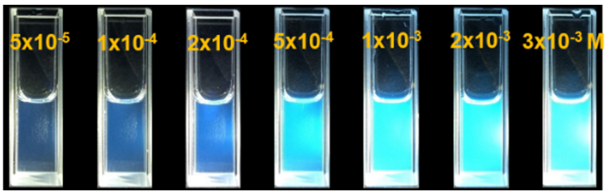	40.70 %<br>(2×10^{-4} M, 0.035 wt.%)<br>34.22 %<br>(3×10^{-3} M, 0.525 wt.%)	This work
Lysine	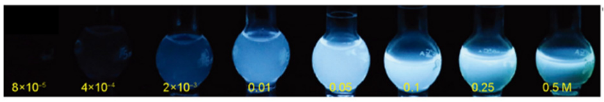	Not reported<br>(8×10^{-5} to 0.5 M)	13
Amino acids	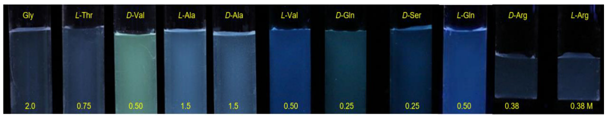	Not reported<br>(0.25 to 2.0 M)	13
Alginate	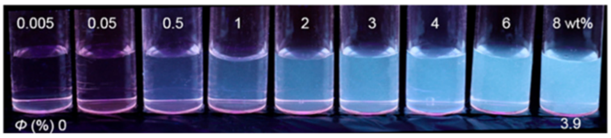	3.9%<br>(8 wt.%)	14

Supplementary Fig. 17. Comparation of the fluorescence performance of CDHis solution with other nonconventional luminophores including concentrated amino acid and alginate solutions.

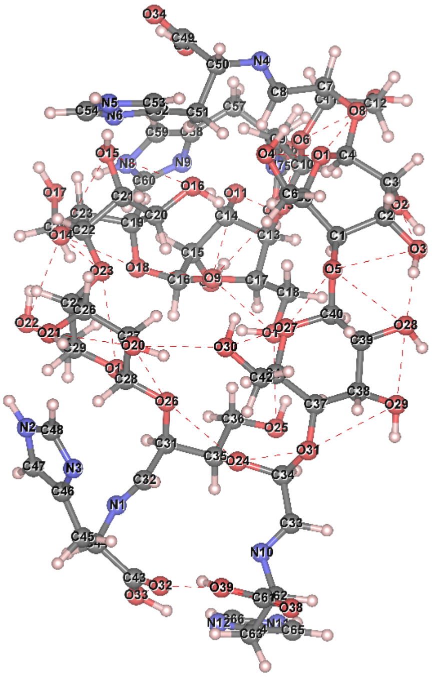

Supplementary Fig. 18. The O−O short contact (smaller than 3.04 Å) in the CD skeleton.

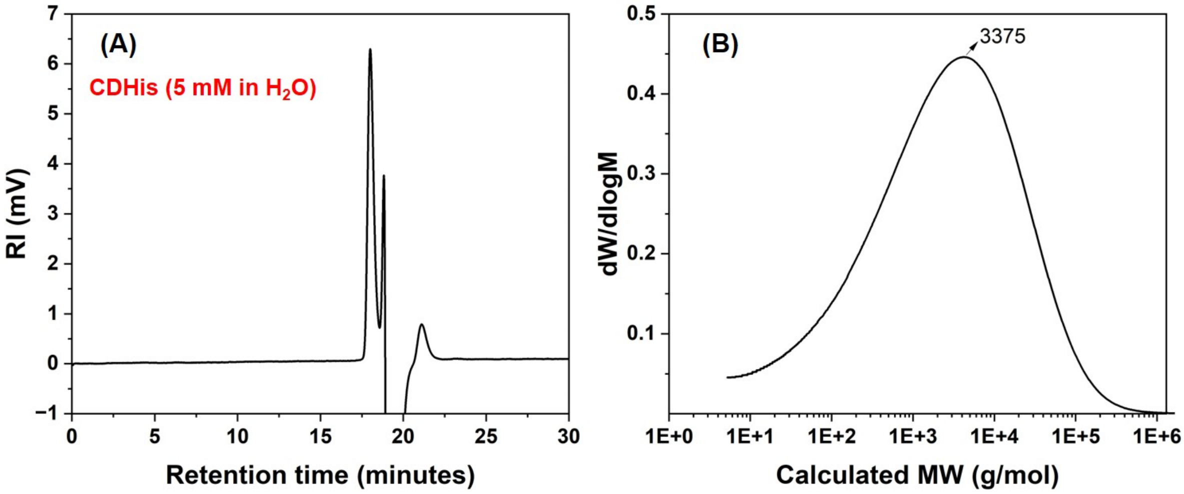

Supplementary Fig. 19. Mass analysis of CDHis solution by gel permeation chromatography (GPC). (A) GPC of CDHis solution (5 mM in H_2O), and (B) the calculated molecular weight for the main peak.

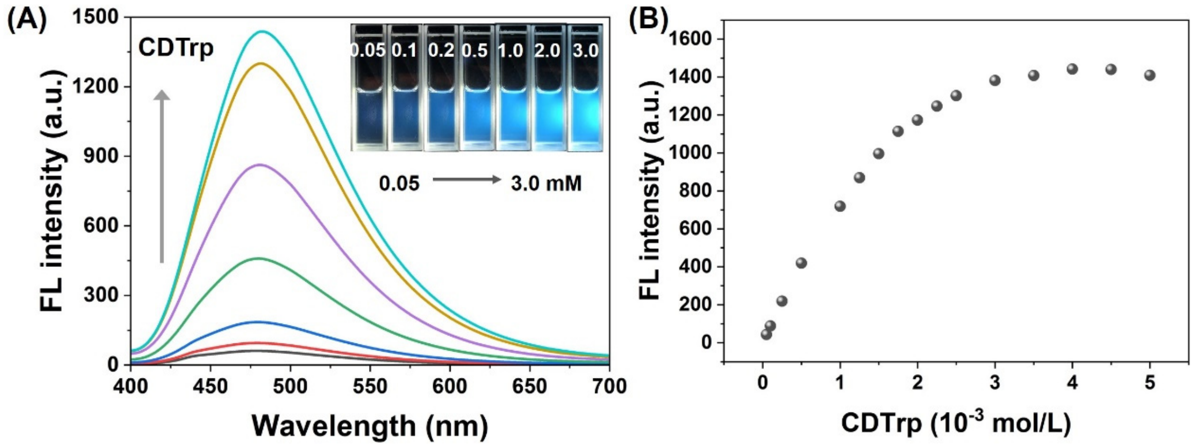

Supplementary Fig. 20. Concentration-dependent fluorescence performance of CDTrp. (A) Concentration-dependent FL spectra of CDTrp solution (inset: photographs of CDTrp solutions taken under 365 nm UV light). (B) Plots of FL intensity vs. CDTrp concentration.

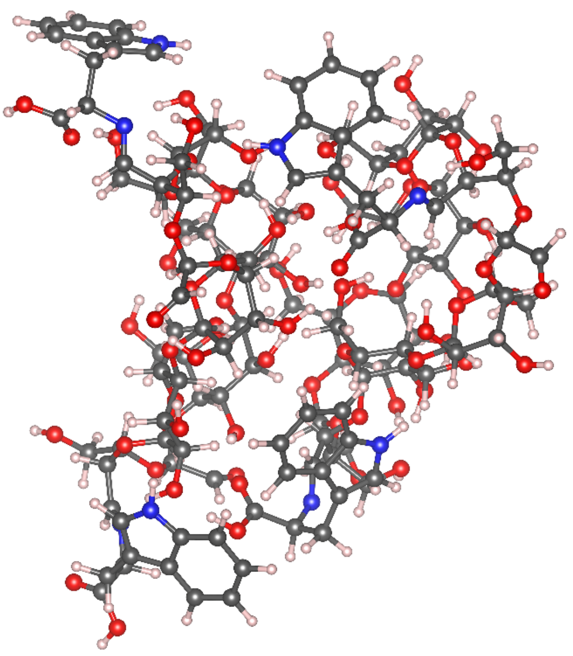

Supplementary Fig. 21. The optimized conformation of CDTrp dimer in H_2O with the binding energy of -30.3 kcal/mol.

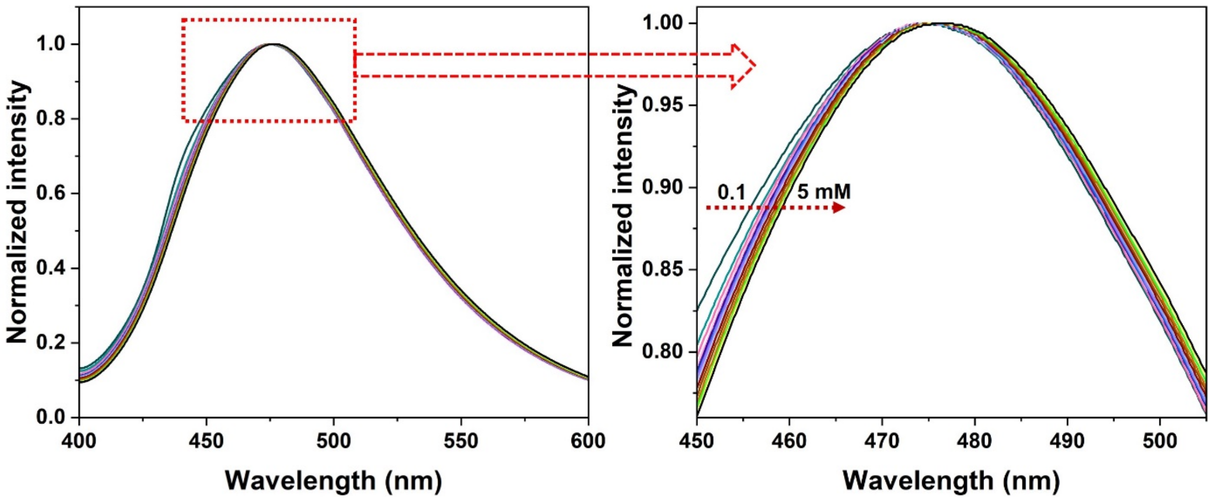

Supplementary Fig. 22. Evolution of fluorescence spectra of CDHis with different concentrations from 0.1 to 5 mM.

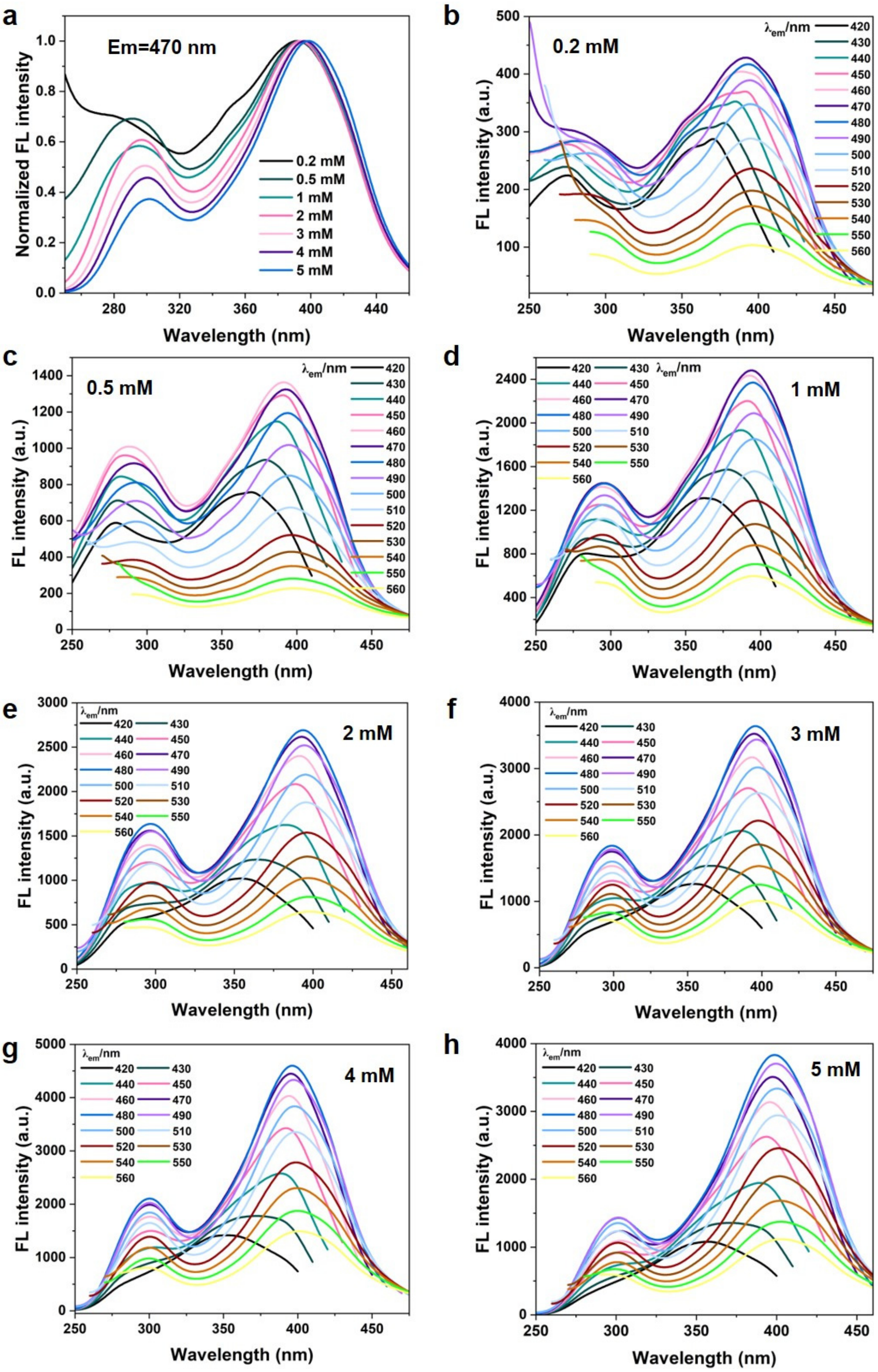

Supplementary Fig. 23. Excitation spectra of CDHis with different concentrations. (a) Excitation spectra (λ_{em} = 470 nm) of CDHis solution at different concentrations from 0.2 to 5 mM. (b-h) The excitation spectra of all the emission bands of CDHis obtained at different concentrations.

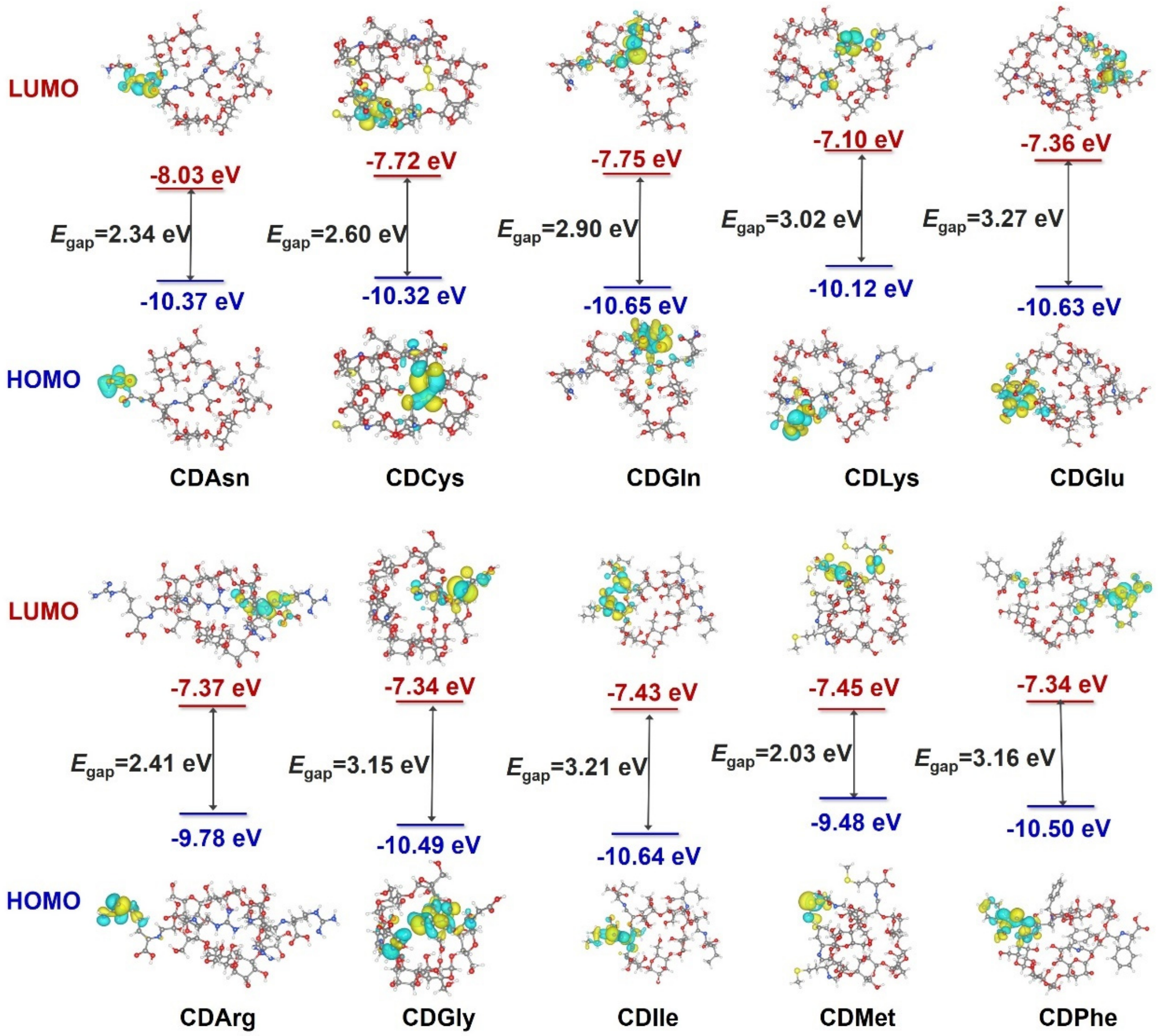

Supplementary Fig. 24. The electron density distribution of HOMO and LUMO levels of all other CDAAs.

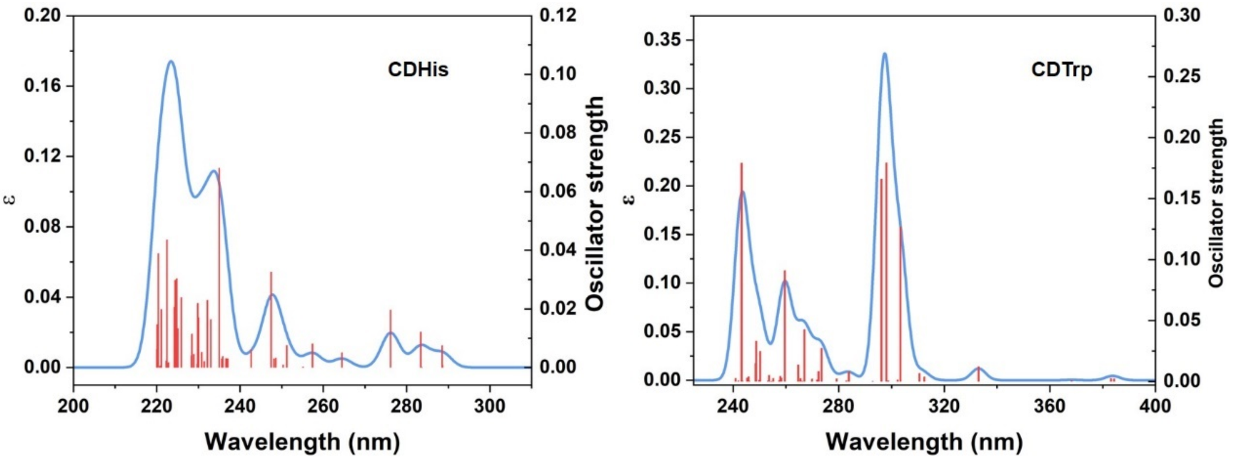

Supplementary Fig. 25. Theoretical absorption spectra of CDHis and CDTrp obtained by TDDFT calculation.

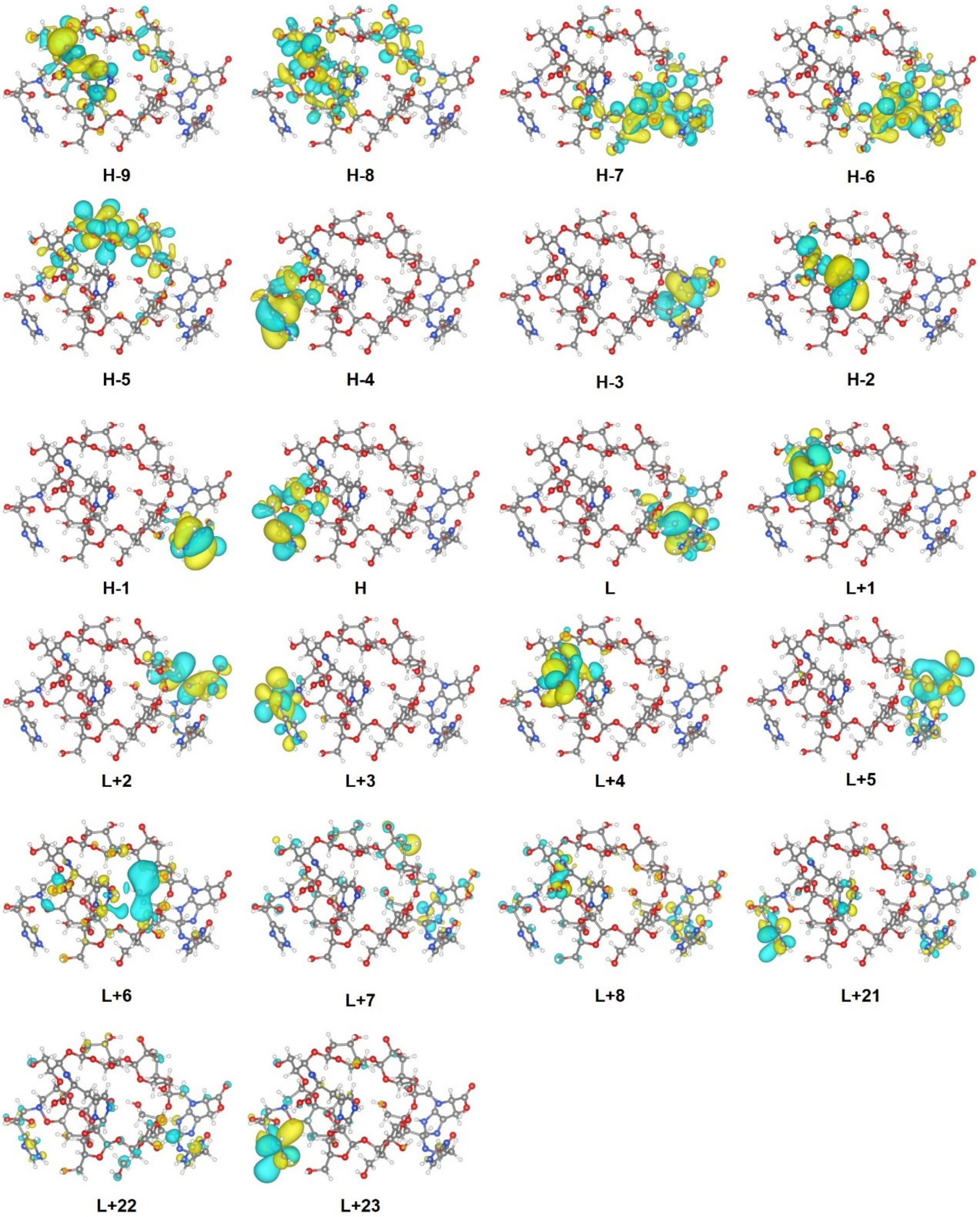

Supplementary Fig. 26. The theoretical orbitals involed in the excitation process of CDHis.

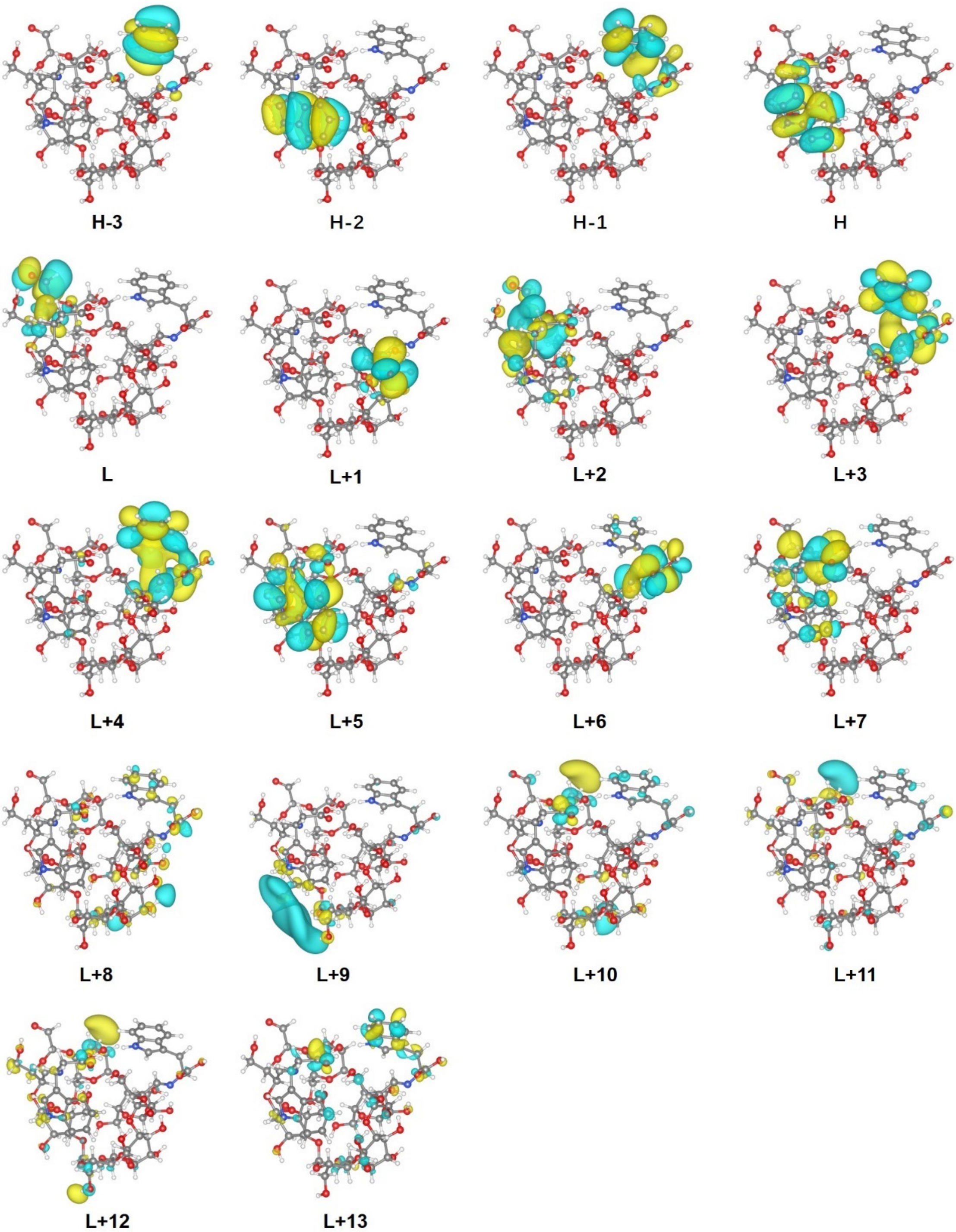

Supplementary Fig. 27. The theoretical orbitals involed in the excitation process of CDTrp.

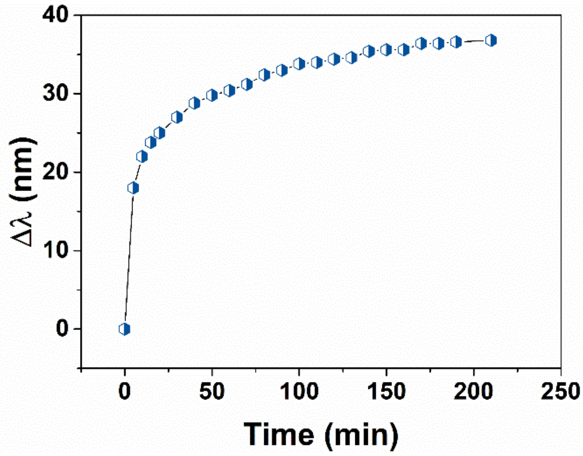

Supplementary Fig. 28. The emission peak shift of CDHis after adding 10 μM CTC.

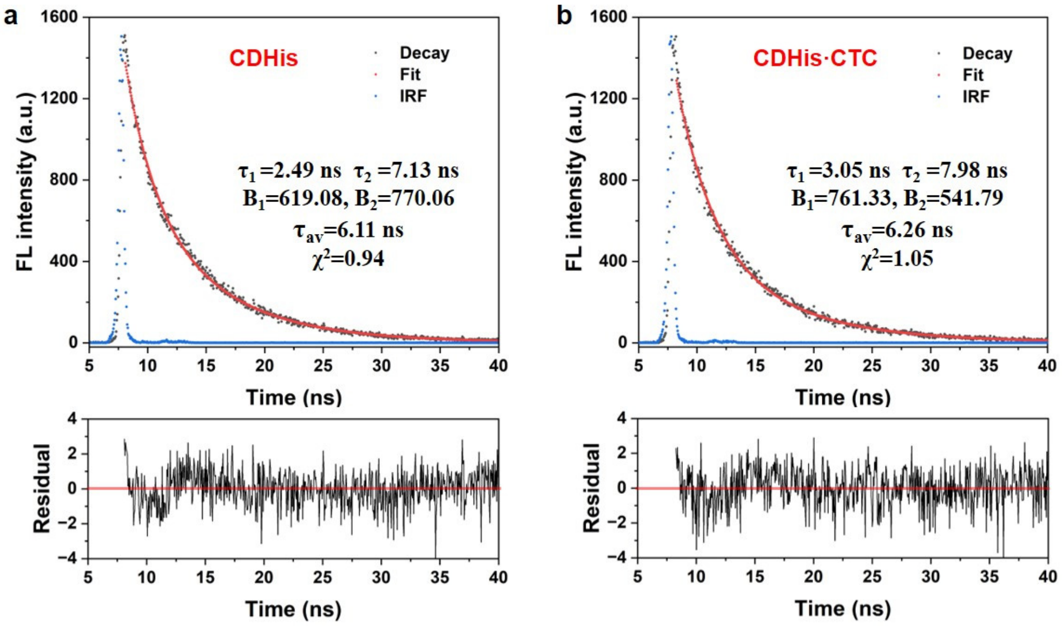

Supplementary Fig. 29. The fluorescence time-resolved decay spectra and fitting results of (a) CDHis and (b) CDHis•CTC complex.

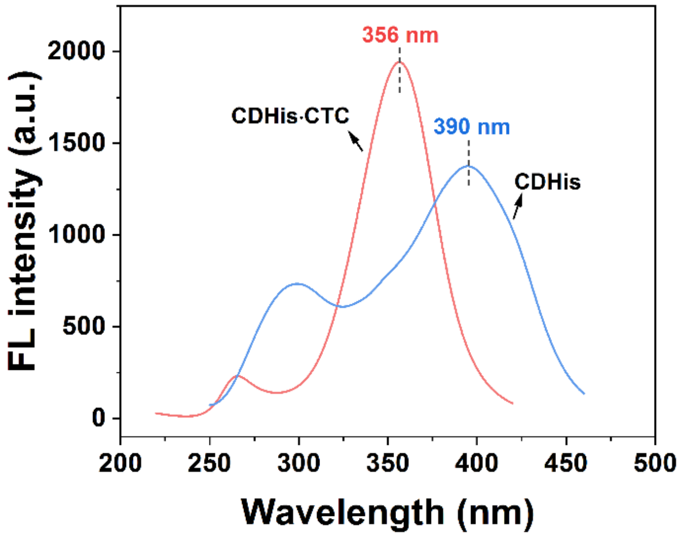

Supplementary Fig. 30. Excitation spectra of CDHis and CDHis•CTC complex.

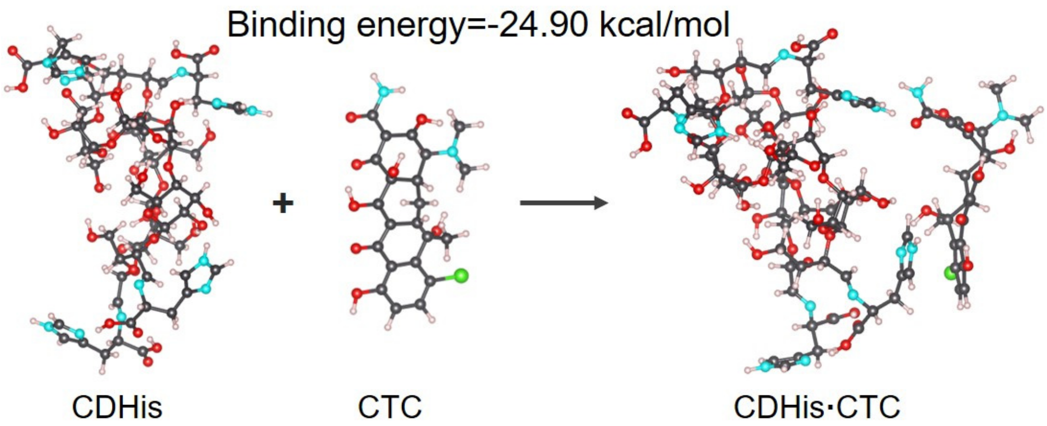

Supplementary Fig. 31. The optimized binding geometry of the CDHis•CTC complex at the B3LYP-D3/6–311G(d)/SMD(water) level of theory.

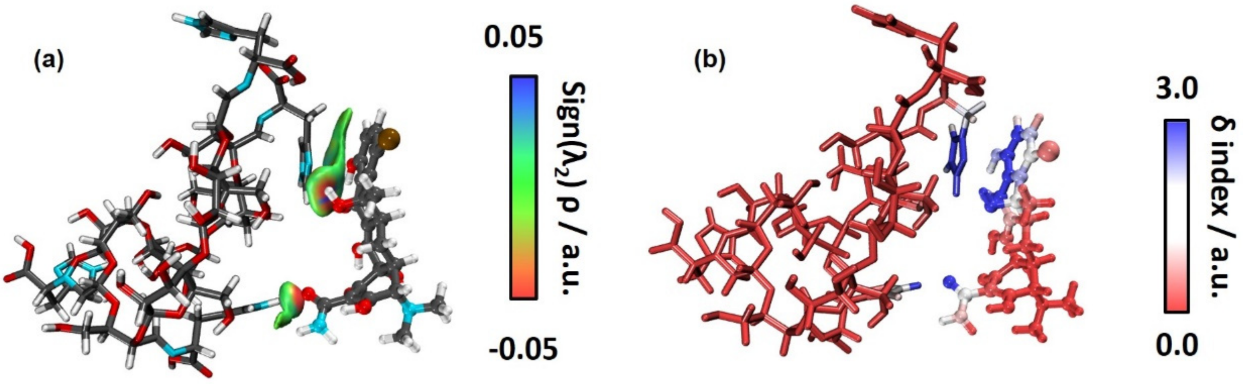

Supplementary Fig. 32. Independent gradient model (IGM) analysis of CDHis•CTC complex. **(a)** δg^{inter} = 0.01 a.u. isosurfaces colored by the sign of $(\lambda_2)\rho$ for the CDHis•CTC complex. The red indicates strong attraction, while the blue indicates strong repulsion. **(b)** The atoms in CDHis (left) and CTC (right) molecules are marked to show their contributions to the complexation. The red indicates low contribution to the complexation, and the blue indicates the high contribution.

The IGM analysis (Supplementary Fig. 32) shows the strong N-H···O hydrogen bonds between imidazole rings of His moiety in CDHis and carbonyl group and hydroxyl group in CTC (red areas in the isosurfaces). The green areas in the isosurfaces indicate the existence of π-π stacking interactions of the imidazole rings in His moiety and aromatic ring in CTC. The atoms of CDHis and CTC are marked according to their contributions to the molecular recognition, which clearly shows that the main contribution derives from the imidazole rings of His moiety in CDHis and aromatic rings and O-containing groups in CTC.

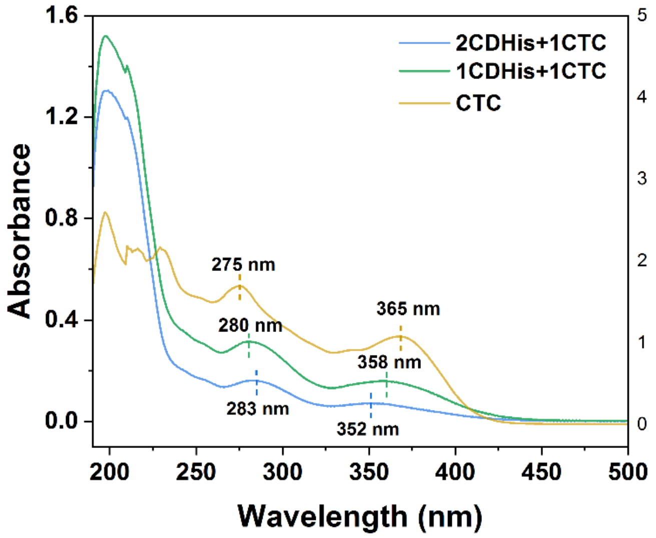

Supplementary Fig. 33. UV absorption spectra of CDHis and CTC systems.

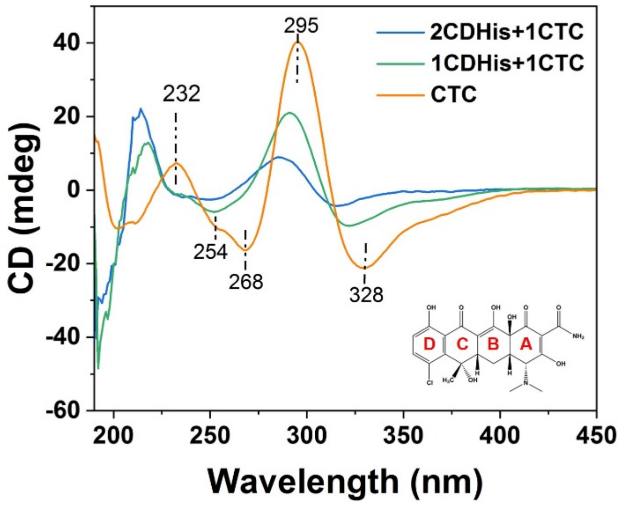

Supplementary Fig. 34. Circular dichroism spectra of CDHis and CTC systems. The BCD-ring chromophores of CTC are responsible for the bands at 328 nm, 295 nm, and 232 nm, while the A-ring chromophore contributes to the 268 nm band[15].

Supplementary Table 1. The excited-state data of CDHis obtained by TDDFT calculation.

State	Excited energy/eV	Wavelength/nm	f	Contributuion	
S2	4.297	288.54	0.0075	H-1→L	87.7%
S4	4.375	283.39	0.0121	H→L+3	96.3%
S5	4.490	276.16	0.0197	H-9→L+1	17.0%
				H-2→L+1	76.0%
S7	4.688	264.46	0.0051	H-7→L	33.5%
				H-6→L	44.6%
				H-1→L	10.9%
S8	4.817	257.40	0.0081	H-3→L+2	95.0%
S10	4.935	251.23	0.0076	H→L+4	21.1%
				H→L+6	15.9%
				H→L+7	18.3%
				H→L+8	26.5%
S14	5.011	247.45	0.0326	H-9→L+1	45.3%
				H-2→L+1	17.0%
				H-2→L+4	20.6%
S23	5.276	235.00	0.0680	H→L+7	12.2%
				H→L+21	11.2%
				H→L+23	24.2%
S43	5.5734	222.46	0.0436	H-6→L+2	10.0%
				H-3→L+5	65.4%
S48	5.6255	220.40	0.0389	H-5→L+2	16.0%
				H-1→L+5	23.6%

Supplementary Table 2. The excited-state data of CDTrp obtained by TDDFT calculation.

State	Excited energy/eV	Wavelength/nm	f	Contributuion	
S1	3.225	384.38	0.0022	H→L	93.7%
S2	3.236	383.16	0.0024	H→L+1	97.4%
S5	3.723	332.99	0.0121	H→L+2	94.9%
S8	4.087	303.33	0.1269	H-1→L+3	77.1%
				H-1→L+4	20.2%
S12	4.160	298.07	0.1794	H→L+5	90.7%
S13	4.186	296.21	0.1659	H-1→L+3	20.3%
				H-1→L+4	75.0%
S19	4.533	273.49	0.0272	H→L+7	90.5%
S23	4.643	267.01	0.0427	H-2→L+2	82.0%s
S28	4.776	259.62	0.0910	H-3→L+3	49.5%
				H-3→L+4	21.1%
				H-1→L+7	14.5%
S39	4.984	248.74	0.0331	H-1→L+7	8.59%
				H-1→L+8	23.6%
				H-1→L+9	13.0%
S47	5.098	243.22	0.1791	H→L+11	31.3%
				H→L+12	21.6%
				H→L+13	10.6%

Supplementary Table 3. Comparison of the CTC detection performance with fluorescent sensors.

**Probe**	**Linear detection range (μM)**	**LOD (μM)**	**Selectivity**	**Ref.**
Red-CDs	5 − 50	0.025	Nonspecific	16
Cd-MOF	10 −1000	3.61	Nonspecific	17
Al-MOF@Mo/Zn-MOF	0.001–53.33	0.046	Nonspecific	18
$CuInS_2$/ZnS QDs	1 – 50	0.46	Specific	19
BSA-AuNCs	0.2 − 10	0.065	Specific	20
**CDHis**	**0 − 20**	**0.012**	**Specific**	**This work**

Red-CDs: red fluorescent carbon dots

MOF: metal organic framework

QDs: quantum dots

BSA-AuNCs: bovine serum albumin stabilized gold nanoclusters

Supplementary References

1. C. Bannwarth, S. Ehlert and S. Grimme., J. Chem. Theory Comput., 15, 1652-1671 (2019).
2. C. Bannwarth, E. Caldeweyher, S. Ehlert, A. Hansen, P. Pracht, J. Seibert, S. Spicher, S. Grimme, WIREs Comput. Mol. Sci., 2020, 11, e01493. DOI: 10.1002/wcms.1493.
3. Tian Lu, Molclus program, Version 1.9.9.9, http://www.keinsci.com/research/molclus.html (accessed 08 03, 2022).
4. Tian Lu, Feiwu Chen, J. Comput. Chem., **33,** 580-592 (2012).
5. Gaussian 16, Revision C.01, M. J. Frisch, G. W. Trucks, H. B. Schlegel, G. E. Scuseria, M. A. Robb, J. R. Cheeseman, G. Scalmani, V. Barone, G. A. Petersson, H. Nakatsuji, X. Li, M. Caricato, A. V. Marenich, J. Bloino, B. G. Janesko, R. Gomperts, B. Mennucci, H. P. Hratchian, J. V. Ortiz, A. F. Izmaylov, J. L. Sonnenberg, D. Williams-Young, F. Ding, F. Lipparini, F. Egidi, J. Goings, B. Peng, A. Petrone, T. Henderson, D. Ranasinghe, V. G. Zakrzewski, J. Gao, N. Rega, G. Zheng, W. Liang, M. Hada, M. Ehara, K. Toyota, R. Fukuda, J. Hasegawa, M. Ishida, T. Nakajima, Y. Honda, O. Kitao, H. Nakai, T. Vreven, K. Throssell, J. A. Montgomery, Jr., J. E. Peralta, F. Ogliaro, M. J. Bearpark, J. J. Heyd, E. N. Brothers, K. N. Kudin, V. N. Staroverov, T. A. Keith, R. Kobayashi, J. Normand, K. Raghavachari, A. P. Rendell, J. C. Burant, S. S. Iyengar, J. Tomasi, M. Cossi, J. M. Millam, M. Klene, C. Adamo, R. Cammi, J. W. Ochterski, R. L. Martin, K. Morokuma, O. Farkas, J. B. Foresman, and D. J. Fox, Gaussian, Inc., Wallingford CT, 2019.
6. C. Jean- Louis, Density- functional theory of atoms and molecules. R.G. Parr and W. Yang, Oxford University Press, New York, Oxford, 1989. IX + 333 pp., International Journal of Quantum Chemistry **47,** 101-101 (1993).
7. K. Burke, Perspective on density functional theory, The Journal of Chemical Physics **136,** 150901 (2012).
8. S. Grimme, S. Ehrlich and L. Goerigk, “Effect of the damping function in dispersion corrected density functional theory,” Journal of computational chemistry, **32,** 1456-65 (2011).
9. Marenich, A. V.; Cramer, C. J.; Truhlar, D. G., Universal solvation model based on solute electron density and on a continuum model of the solvent defined by the bulk dielectric constant and atomic surface tensions. The Journal of Physical Chemistry B, **113,** 6378 (2009).
10. Ge, X. et al. Inclusion complexation of chloropropham with beta-cyclodextrin: Preparation, characterization and molecular modeling. Spectrochimica Acta Part a-Molecular and Biomolecular Spectroscopy **81,** 397-403 (2011).
11. Lou, C., Tian, X., Deng, H., Wang, Y. & Jiang, X. Dialdehyde-beta-cyclodextrin-crosslinked carboxymethyl chitosan hydrogel for drug release. Carbohydrate Polymers **231,** 115678 (2020).
12. Kobayashi, M. et al. CYCLODEXTRIN DIALDEHYDE PREPARED BY PERIODATE-OXIDATION. Agricultural and Biological Chemistry **52,** 2695-2702 (1988).
13. Chen, X. *et al.* Prevalent intrinsic emission from nonaromatic amino acids and poly(amino acids). *Sci. China Chem.* **61**, 351-359, (2018).
14. Dou, X. *et al.* Clustering-Triggered Emission and Persistent Room Temperature Phosphorescence of Sodium Alginate. *Biomacromolecules* **19**, 2014-2022, (2018).
15. Lambs, L., Decocklereverend, B., Kozlowski, H. & Berthon, G. METAL ION-TETRACYCLINE INTERACTIONS IN BIOLOGICAL-FLUIDS .9. CIRCULAR-DICHROISM SPECTRA OF CALCIUM AND MAGNESIUM COMPLEXES WITH TETRACYCLINE, OXYTETRACYCLINE, DOXYCYCLINE, AND CHLORTETRACYCLINE AND DISCUSSION OF THEIR BINDING MODES. Inorg. Chem. **27,** 3001-3012 (1988).
16. Li, L. et al. Red fluorescent carbon dots for tetracycline antibiotics and pH discrimination from

aggregation-induced emission mechanism. Sensor. Actuat. B-Chem. 332, 129513 (2021).
17. Li, Q.-Q. et al. Multiple fluorescence response behaviors towards antibiotics and bacteria based on a highly stable Cd-MOF. J. Hazard. Mater. 423, 127132 (2022).
18. Li, C. et al. A 3D hierarchical dual-metal–organic framework heterostructure up-regulating the pre-concentration effect for ultrasensitive fluorescence detection of tetracycline antibiotics. J. Mater. Chem. C 8, 2054-2064 (2020).
19. Chen, X. et al. Dual-mode turn-on ratiometric fluorescence sensor based on carbon dots and CuInS2/ZnS quantum dots for detection of chlorotetracycline. Spectrochim. Acta. A. 270, 120851 (2022).
20. Meng, L., Lan, C., Liu, Z., Xu, N. & Wu, Y. A novel ratiometric fluorescence probe for highly sensitive and specific detection of chlorotetracycline among tetracycline antibiotics. Anal. Chim. Acta. 1089, 144-151 (2019).
